# The TreadWheel: A Novel Apparatus to Measure Genetic Variation in Response to Gently Induced Exercise for Drosophila

**DOI:** 10.1371/journal.pone.0164706

**Published:** 2016-10-13

**Authors:** Sean Mendez, Louis Watanabe, Rachel Hill, Meredith Owens, Jason Moraczewski, Glenn C. Rowe, Nicole C. Riddle, Laura K. Reed

**Affiliations:** 1 Department of Biological Sciences, University of Alabama, Tuscaloosa, AL, United States of America; 2 Department of Biology, University of Alabama at Birmingham, Birmingham, AL, United States of America; 3 Department of Medicine, University of Alabama at Birmingham, Birmingham, AL, United States of America; National Center for Geriatrics and Gerontology, JAPAN

## Abstract

Obesity is one of the dramatic health issues affecting developed and developing nations, and exercise is a well-established intervention strategy. While exercise-by-genotype interactions have been shown in humans, overall little is known. Using the natural negative geotaxis of *Drosophila melanogaster*, an important model organism for the study of genetic interactions, a novel exercise machine, the TreadWheel, can be used to shed light on this interaction. The mechanism for inducing exercise with the TreadWheel is inherently gentle, thus minimizing possible confounding effects of other stressors. Using this machine, we were able to assess large cohorts of adult flies from eight genetic lines for their response to exercise after one week of training. We measured their triglyceride, glycerol, protein, glycogen, glucose content, and body weight, as well as their climbing ability and feeding behavior in response to exercise. Exercised flies showed decreased stored triglycerides, glycogen, and body weight, and increased stored protein and climbing ability. In addition to demonstrating an overall effect of TreadWheel exercise on flies, we found significant interactions of exercise with genotype, sex, or genotype-by-sex effects for most of the measured phenotypes. We also observed interaction effects between exercise, genotype, and tissue (abdomen or thorax) for metabolite profiles, and those differences can be partially linked to innate differences in the flies' persistence in maintaining activity during exercise bouts. In addition, we assessed gene expression levels for a panel of 13 genes known to be associated with respiratory fitness and found that many responded to exercise. With this study, we have established the TreadWheel as a useful tool to study the effect of exercise in flies, shown significant genotype-specific and sex-specific impacts of exercise, and have laid the ground work for more extensive studies of how genetics, sex, environment, and aging interact with exercise to influence metabolic fitness in Drosophila.

## Introduction

Obesity is a health concern that has reached epidemic proportions and has been the subject of international legislation in an attempt to curb its prevalence [1]. The leading hypothesis for the primary cause of the recent surge in obesity in the US is increases in both sedentary behavior and caloric intake [2]. Given that the US spent 190 billion dollars on obesity-associated medical expenses in 2005 and that those costs are projected to rise [3], novel approaches to reduce obesity are needed to lessen the financial and medical costs on society. Current treatment options for obesity include surgical interventions such as gastric bypass and lifestyle interventions such as weight-loss through changes in exercise and diet. Exercise induced weight-loss has almost no inherent risks and can be effective in reducing the severity of psoriasis [4], increasing insulin sensitivity [5], and acting as a preventative measure for conditions such as cardiovascular disease [6]. Thus, regular exercise and moderate activity levels are considered important components of maintaining a healthy lifestyle [7].

Despite the popularity of exercise as a treatment for obesity, it is not as universally effective as many presume[8,9]. Many other factors such as genetic and sex differences interact with exercise to influence its impact on obesity; yet the impact of sex and genetic variation on the physiological effects of exercise is poorly understood [10]. Experiments in humans demonstrate that genetic background influences the effects of exercise on metabolism [11–13], and studies in mice have shown the same for body composition [14]. Moreover, exercise resistance is an exciting new field focused on, individuals who may be programmed—genetically or epigenetically—to have a weak or absent metabolic response to exercise [15]. Despite this progress, only a handful of potential candidate genes predicting exercise response have been identified, and follow-up research has had limited success [16]. Thus, it remains unclear if single genes, epistatic interactions, epigenetics, or a combination of these factors control exercise response. The lack of progress in this area is at least partially due to the limitations of using human subjects, as it is difficult to control for genetic background and environmental factors. Fortunately, these issues can be overcome by studying genes related to exercise in the *Drosophila melanogaster* model.

The fruit fly *Drosophila melanogaster* is an excellent model organism for studying the genetics of exercise. The *D*. *melanogaster* genome contains many genes homologous with those of humans [17], and energy related pathways are highly conserved between Drosophila and humans [18]. Drosophila are inexpensive and easy to maintain in large numbers under tightly controlled environmental conditions, allowing for larger sample sizes and thus for greater statistical power than is possible in other model organisms. There are also are a number of specialized tools for assessing Drosophila behavior such as feeding [19]. In addition, there are many genetic resources available in Drosophila, such as the DGRP2 (Drosophila Genetics Reference Panel 2), a fully sequenced set of 200 inbred, genetically diverse lines [20,21]. Study populations such as the DGRP2 are useful for QTL (quantitative trait loci) mapping and GWAS (genome wide association studies) and contribute to the power of the Drosophila model.

Although still a burgeoning field, several Drosophila exercise experiments already have demonstrated behavioral and physiological responses to exercise. These experiments use the Power Tower, a device that utilizes the fly’s inherent negative geotaxis, repeatedly dropping an enclosure of the flies, knocking the flies to the base, and inducing the flies to climb. In these experiments, climbing ability, a measure of physical fitness, was examined after an endurance exercise regime, and the response was affected by the factors of diet and age [22,23]. Thus, these studies firmly established the use of Drosophila as a model for exercise. However, the repeated physical impact of the flies against the base of their enclosure in the Power Tower is physically intense and stimulates sustained activity in the flies that could be associated with behavioral or physical stress-related effects [24]. Therefore, there is a need for a complementary approach to test for gently induced exercise to better understand how exercise-type influences physiological response.

In this study, we utilize a novel combination of techniques to obtain data regarding the effects of exercise on adult fly metabolism and fitness, while minimizing any additional physical stresses as induced by the Power Tower method. Instead of dropping the enclosures, the TreadWheel uses slow end-over-end rotations of the fly enclosures to induce easily observed, continuous climbing by negative geotaxis. We explore the effects of exercise with the TreadWheel on a variety of outcome measures including body weight, stored metabolite levels, physical fitness (climbing performance), feeding behavior, and gene expression to evaluate this novel exercise system. We find that there are significant differences in the various outcome measures between flies experiencing the TreadWheel exercise regime and control, unexercised, flies. Moreover, the effects of exercise on these outcome measures varied by genotype, and these genotypic differences in exercise response are partially explained by innate differences among the lines in their persistence in maintaining activity during exercise bouts. These results support the use of the TreadWheel as a complementary method to the Power Tower, a model for the biology of exercise induction in Drosophila and illustrate its potential for studies on the impact of genetic variation on exercise response.

## Materials and Methods

The data presented in this manuscript were generated in two parallel studies at the University of Alabama in Tuscaloosa (Study A) and the University of Alabama at Birmingham (Study B). Methodologies used by the two studies were very similar but differed in some specific details as noted below. Data from the two studies were analyzed independently.

### Exercise conditions

**Fig 1:**
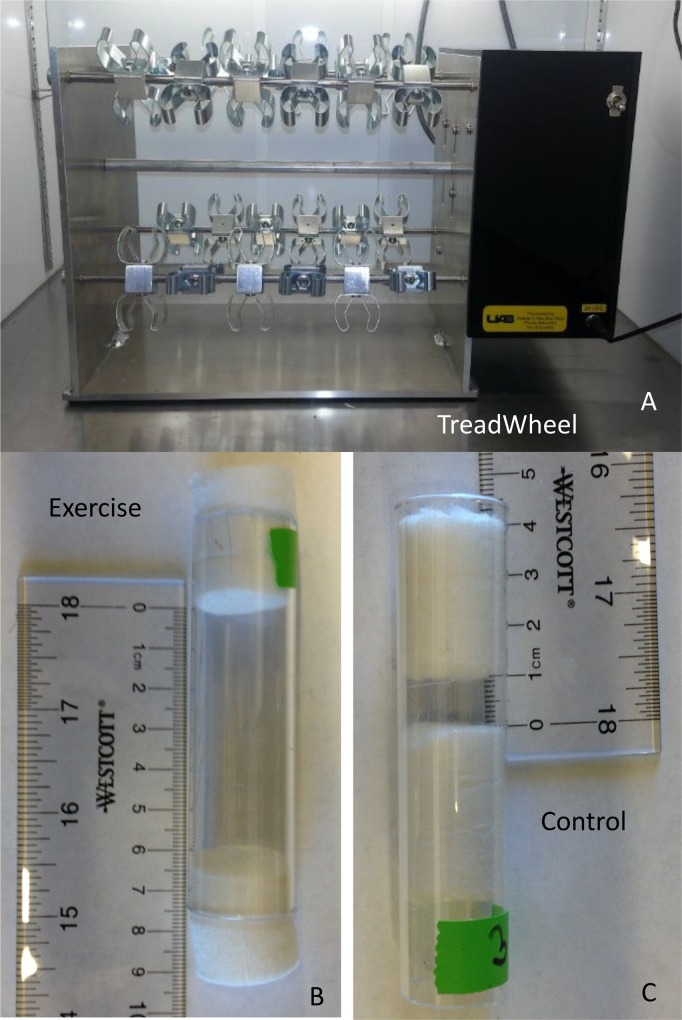
The TreadWheel exercise machine. A. The TreadWheel holds a maximum of 48 vials, and each clamp is removable. The unit features an adjustable rotation speed, with these experiments using 4RPM. B. The distance between the plugs at the top and bottom of the vial for each vial was adjusted to 6cm for the exercised flies and to 1cm for the control flies. These vials were then clipped into the TreadWheel for the exercise regime.

Two identical TreadWheel machines were built in the UAB machine shop from the prototype first built by S. Mendez and used at the two research sites (S1 File). The TreadWheel has capacity for 48 fly vials held on four axels with metal clips (Fig 1A) and is powered by a variable speed electric motor. It is compact enough to fit into a standard Drosophila incubator. When loaded and running, the TreadWheel slowly rotates fly vials lengthwise, so that the gravitational top of the vial constantly changes. As is readily observed, the slowly rotating vial thus provides a continuous stimulus to climb due to the flies’ innate negative geotaxis, the behavioral tendency to climb upwards whenever possible. For the experiments reported here, the rotation speed of the TreadWheel was set to four rotations per minute (RPM).

The exercise regime consisted of up to two hours of exercise each day for five days. The exercise period occurred at the same time each day. Study A used a training regime that consisted of four short exercise bouts separated by five-minute rest periods, with the bout durations gradually increasing from 15 to 20 minutes over the course of the five days of training (S1 Table). Study B used one two-hour continuous exercise bout without rest periods, each day for five days. In Study A, the flies were placed in their empty exercise vials only for the exercise time period and were returned to fresh food vials after each daily exercise period (which allowed them to engage in normal movement and feeding behavior between exercise periods), while in Study B, the food vials were used as the exercise vials. Exercise vials contained six centimeters of space for movement by the flies (Fig 1B). Controls in Study A consisted of vials loaded on the TreadWheel constraining the flies to one centimeter of space during the exercise bout (Fig 1C); they thus experienced the rotation with limited mobility. Similar controls are used in Power Tower studies [22–24]. In contrast, the controls in Study B were flies that were placed in front of the TreadWheel, allowing them to exhibit normal movement throughout the experimental period while being exposed to the mild noise and vibrations from the TreadWheel.

In Study A, male and female mated 5–7 day old flies exercised in single sex groups of 10 per vial, while in Study B male virgin 3–5 day old flies exercised in groups of 20 per vial. All flies were maintained at 25°C and 50% humidity with 12h light/dark cycles for two generations prior to and during the experiment.

### Drosophila lines and husbandry

Study A used the canonical Drosophila lab strains acquired from the Bloomington Stock Center and fellow Drosophila labs, *y*^*1*^*w*^*67c23*^ (Bloomington 6599), *y*^*1*^*w*^*1*^ (Bloomington 1495), *w*^*1118*^ (Janis O'Donnell, University of Alabama), and ORE-R P2 (Edwin Stephenson, University of Alabama). Study B used lines 307, 315, 380, and 852 from the DGRP2, a wild-type population consisting of over 200 inbred lines [20,21]. The four DGRP2 Drosophila lines were selected as they represent a diverse group of mitochondrial efficiencies previously determined by Dr. Maria DeLuca (UAB, unpublished result). All flies were maintained on a standard cornmeal-molasses food (by weight 5.28% cornmeal, 1.05% yeast, 0.56% agar, 87.03% water, 4.37% molasses, 1.15% Tegosept, 0.55% Propionic acid) seeded with live yeast (*e*.*g*. [25]).

### Metabolic phenotypes

Samples were collected after a 24-hour (Study A) or 48-hour (Study B) recovery period following the five-day exercise regime and stored at -20°C. Fly wet weights were measured on individual flies (Study A) or on groups of five flies (Study B) using a high precision balance. Measurements of metabolite pools were conducted on whole flies in groups of ten in Study A and separately on thoraces and abdomens in groups of five in Study B. In study A, the distinct metabolite pools were measured on separate samples for the glucose, triglyceride/protein, and glycogen phenotypes, while Study B used the extract from the same homogenate from a given sample for all metabolite measurements (S2 File). Total protein content was estimated using the Bradford method [26,27] (Study A) and the Lowry method (Study B) [28] (S2 File). Glucose content, as a surrogate measure of circulating trehalose, was determined by enzymatic digestion of trehalose to glucose then measured by absorbance using the Sigma Glucose Assay Kit (GAGO20) as described in [25] and S2 File. Triglyceride concentrations was determined by absorbance using the Sigma Serum Triglyceride Determination Kit (TR0100), and glycerol concentrations (Study B) were determined by absorbance using the Sigma Free Glycerol reagent (F6428, [25], S2 File). Glycogen levels (Study A) were measured using the Sigma-Aldrich Glycogen Assay Kit (MAK016, S2 File).

### Motivation

We quantified how long it took each line to cease exercising by visually inspecting the flies for the first four days of the regime in Study B and recorded when ~50% of the flies from each genotype ceased whole body locomotion activity (low levels of activity were maintained in some individuals). Follow-up experiments confirmed these visual observations with video recordings. In subsequent analyses, we categorized the four lines in Study B into two groups, low and high motivation.

### Feeding behavior

A modified version of the CAFE (Capillary Feeder) assay was used to assess the innate differences in feeding behavior among the lines in Study B and how exercise influenced feeding behavior [29]. Briefly, pipette tips were inserted through foam vial tops, such that the glass capillaries can be stably held through the tops, and a nutrient solution is provided through the capillaries to the flies. Food consumption is measured by recording the drop in liquid levels in the capillary. On the second day of exercise, five male virgin flies were placed into each feeding vial with 8μl of 10% sucrose, 5% yeast solution loaded into each capillary. Flies were acclimatized to the CAFE environment for one day. After the third day of exercise, the capillaries were refilled, and the amount of nutrient solution consumed by the flies after 8hrs was quantified using a ruler.

### Climbing ability

In Study A, negative geotaxis assays similar to those described in Gargano et al.[30] were used to assess climbing ability after a 24-hour recovery period following five days of exercise (S1 Fig). In Study B, the negative geotaxis assay was performed the day before the exercise regime and again immediately following the third day of exercise. Ten (Study A) or twenty (Study B) flies were loaded into each empty vial and placed in a rack in front of a one-centimeter grid (Study A) or a light box (Study B). Flies were moved to the bottom of the vial by tapping the vial on the counter top. After a four second (Study A) or two second (Study B) delay, a camera photographed the vials to record how high the flies could climb (S1 Fig). In Study A, we quantified the height of each fly to nearest half centimeter. In Study B, a climbing index was calculated by dividing each vial into four quadrants, counting the number of flies in each quadrant, multiplying by the point value to each quadrant from one (bottom) to four (top), summing those values then dividing the by the total number of flies in the vial, and then the final climbing score for a given vial of flies was the average of four repeated assays run in short succession.

### Gene expression

Two lines, one high activity and one low activity (DGRP 315 and 380) from Study B were assayed for gene expression levels in a panel of exercise and mitochondrial function associated genes [31] using Q-RT-PCR (S2 Table). RNA was isolated from 20 virgin male flies frozen at -80°C 48 hours following the completion of the exercise regime, using Trizol reagent (Thermo Fisher Scientific) in three independent biological replicates. RNA was subjected to reverse transcription using a High Capacity cDNA synthesis kit (Thermo Fisher Scientific). Q-RT-PCR was performed on the cDNA with gene specific primers in the presence of the fluorescent dye SYBR green (BioRad). The average expression of three house-keeping genes (*RBM34*, *RPL32*, *TBP*) was used to determine ΔCT for the target genes of interest; ΔCT values were then converted back to a linear scale of relative expression for statistical analysis. Full gene names and primers used for Q-RT-PCR are listed in S2 Table.

### Statistical analyses

Statistical analyses were performed using JMP Pro 11. Study A and Study B data were analyzed independently. Glucose concentrations were log transformed for normality prior to statistical analysis; the other phenotypes required no transformation. We checked for the contributions of various experimental variables (*e*.*g* exercise treatment, genetic line, tissue, sex, motivation, and their interactions) on the measured phenotypes (weight, metabolite levels, feeding behavior) using analysis of variance (ANOVA). Block effects were included in the ANOVA as co-factors when there was block structure to the experimental design and were only found to be significant in the Study A negative geotaxis assay (p<0.0001). *Post hoc* tests for pairwise contrasts were performed using a Student's t-test. S3–S6 Tables give sample sizes, phenotype means, and standard errors (SE) for each measured phenotype stratified by experimental treatments (e.g. exercise treatment, genetic line, tissue, sex, motivation, and feeding behavior). Treatment combinations with two or fewer replicates were excluded from statistical analyses. Multiple testing corrections (Bonferroni) are noted where used.

## Results and Discussion

The TreadWheel is a novel, high throughput device designed to gently induce exercise by the slow rotation (4RPM) of the fly enclosure, stimulating adult flies to walk continuously toward the top of the enclosure due to their innate negative geotaxis. The overall results of this analysis are that the TreadWheel is an effective method to induce exercise in adult Drosophila, and it shows substantial impacts on metabolic traits, climbing performance, and mitochondrial gene expression (see below). The machine easily fits standard sized Drosophila vials with a simple clip-in mechanism, and up to 48 vials of flies can be treated simultaneously (Fig 1A). The TreadWheel also fits inside of a standard Drosophila incubator to allow for the control of other environment conditions during experiments.

The protocols we present here give two alternatives for a five-day exercise regime: one using short bouts separated by short rest times over approximately two hours daily with a gradual increase in bout duration over the week (Study A, S1 Table) and a second with a continuous two-hour bout of moderate induced exercise daily (Study B). Controls either consisted of enclosures with restricted space for movement loaded on the TreadWheel (Fig 1C) or enclosures set immediately next to the TreadWheel to control for noise and vibrations. The results generated by the TreadWheel allows for new opportunities to analyze how gently induced exercise interacts with other factors (*e*.*g*. diet, age, genotype) to modify physiology in a model system of human physiology, Drosophila.

While there are a number of minor differences in the protocols used in Study A and Study B, the results were largely congruent. For many phenotypes, Study A was more robust than Study B in both sample size (S3–S6 Tables) and in the total amount of exercise the flies actually engaged in (see methods and motivation results). Thus, in the situations when Study B fails to find the significant effects of exercise seen in Study A such as for protein, triglycerides, and weight, these differences in study robustness are the likely explanation. However, the trends in Study B are always consistent with the findings in Study A, indicating that the TreadWheel gives consistent results.

### Gently induced exercise on the TreadWheel influences metabolic and fitness phenotypes

Several key metabolites are known to change in response to exercise in other systems [32,33], and changes in weight as a result of a change in caloric intake or exercise are often accompanied by a change in body composition as well as metabolite profile [34]. Thus, we assayed the effect of exercise, innate motivation, and feeding behavior on metabolic profiles to demonstrate the effectiveness of the Treadwheel.

**Fig 2:**
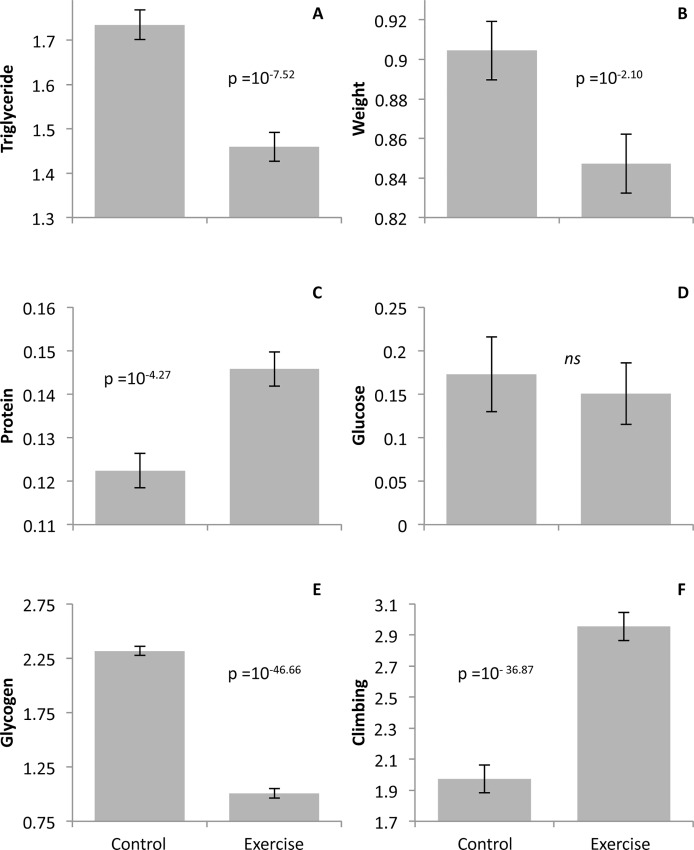
Significant effect of exercise on metabolic traits and climbing ability. Data presented for Study A averaged across all genotypes and both sexes representing a per fly equivalent. Flies experiencing exercise showed a significant decrease in A. triglycerides (0.25 mg/mg protein), B. weight (mg), and E. glycogen (0.00002 mg/mg protein). C. Protein content (0.2 mg/fly) and F. climbing ability (cm) both showed a significant increase in the exercised group relative to controls. D. Glucose content (0.008 mg/ mg protein) was unchanged by the exercise treatment. Error bars indicate SE.

The first major finding of this study is that gently induced exercise on the TreadWheel induces substantial phenotypic effects. On average across all four genotypes and both sexes, the exercise treatment showed a highly significant decrease in fly weight, triglyceride content, and glycogen content as well as an increase in protein content (Fig 2, Tables 1 and 2). The decrease in weight and triglycerides are consistent with the common findings that exercise in obese individuals can reduce these phenotypes [35–39]. Previous studies in humans have indicated that whole body protein synthesis can be increased following exercise, and our flies showed a similar overall increase in protein. However, from these data alone, we cannot determine whether the increased protein was due to increased synthesis or other effects such as decreased catabolism [40]. We might predict that the protein levels would increase especially in the thorax which houses the locomotor muscles used in the induced exercise. Instead, we found no significant effect of exercise on thorax protein (p = 0.184) in Study B. This finding suggests that the whole body exercise response of protein levels in the organism may operate independently of physiological effects on the muscles used is the specific exercise.

**Table 1:** Effects of Line, Sex, Exercise, and their interactions on metabolic and climbing traits.

Phenotype	Line	Sex	Treatment	Line * Sex	Line * Treatment	Sex * Treatment	Line * Sex * Treatment
climbing	**<0.0001**	**<0.0001**	**<0.0001**	**<0.0001**	**<0.0001**	**<0.0001**	**<0.0001**
glucose	**<0.01**	**<0.0001**	*ns*	*ns*	**<0.01**	**<0.01**	*ns*
glycogen	**<0.0001**	**<0.0001**	**<0.0001**	**<0.0001**	**<0.0001**	**<0.0001**	**<0.0001**
protein	**<0.001**	**<0.0001**	**<0.0001**	**<0.0001**	**<0.0001**	**<0.01**	**<0.0001**
triglyceride	**<0.0001**	**<0.0001**	**<0.0001**	**<0.0001**	**<0.0001**	**<0.01**	**<0.01**
weight	**<0.001**	**<0.0001**	**<0.01**	<0.01	**<0.0001**	<0.05	*ns*

ANOVA analysis, Bold indicates significance at a Bonferroni level, *ns*–not significant. Data from Study A.

**Table 2:** Effects of Line, Tissue, Exercise, and their interactions on metabolic traits and feeding behavior.

Phenotype	Line	Tissue	Treatment	Line * Tissue	Line * Treatment	Tissue * Treatment	Line * Tissue * Treatment
CAFÉ	*ns*	-	<0.01	-	<0.01	-	-
glucose	**<0.0001**	**<0.0001**	*ns*	**<0.01**	**<0.01**	*ns*	*ns*
glycerol	**<0.0001**	**<0.01**	*ns*	**<0.01**	**<0.001**	*ns*	<0.05
protein	**<0.0001**	<0.05	*ns*	**<0.0001**	*ns*	*ns*	**<0.0001**
triglyceride	**<0.01**	<0.05	*ns*	*ns*	*ns*	*ns*	*ns*
weight	**<0.0001**	-	*ns*	-	*ns*	-	-

ANOVA analysis, Bold indicates significance at a Bonferroni level, *ns*–not significant. Data from Study B.

Glycerol levels were measured separately from triglycerides due to glycerol's involvement in the glycerol-3-phosphate shuttle mechanism, an essential part of the glycolysis pathway [41]. A previous human study has shown that endurance athletes have significantly increased glycerol levels compared to untrained individuals [42]; however, we did not find that glycerol levels were affected by exercise in our study (Fig 2, Tables 1 and 2). We also assessed glucose since increased circulatory glucose levels are associated with sedentary lifestyles in humans [43]; however, we found that glucose levels were not affected by exercise in our study (Fig 2, Tables 1 and 2).

Climbing performance significantly increased relative to controls after a week of exercise in Study A (Fig 2, Table 1), while we also observed that over the age span of the flies used in these experiments, climbing performance also improved with age independent of exercise in Study B (see below). These observations are consistent with the finding that exercise regimes usually lead to an improved ability to perform a task [44].

These results confirm that the Treadwheel indeed induces alterations in fly behavior (*i*.*e*. exercise) and that this behavior leads to physiological changes, similar to what has been reported in other species.

As might be anticipated, variables other than exercise, such as genotype, sex, and tissue type assayed (thorax vs. abdomen), also showed highly significant effects on most phenotypes (Tables 1 and 2). For example, in Study A, sex was a significant predictor for all phenotypes examined, illustrating the importance of carrying out experiments with animals of both sexes. In addition, genotype (“Line” in Tables 1 and 2) had a significant impact on all phenotypes assayed with the exception of feeding behavior in Study B. These results illustrate the complexity in determining the impact of exercise on physiological traits.

Our study also highlights the importance of interaction effects. Genetic line interacted significantly with sex for all phenotypes except glucose content in Study A (Table 1), demonstrating that in order to predict these phenotypes, both genetic background and sex have to be taken in to account. In Study B, genetic line interacted with tissue type significantly for glucose, glycerol, and protein levels, illustrating that metabolite levels do not simply depend on the tissue assayed, but that genetic background influences these levels as well (Table 2). Together, these significant main effects and interactions among non-exercise factors indicate the complexity of biological factors into which we are introducing the added variation of an exercise treatment. Both main effects and interaction effects have to be considered in order to gain a better understanding of the exercise responses induced by the TreadWheel.

### Genetic, sex, and tissue effects interact with exercise to influence phenotype

**Fig 3:**
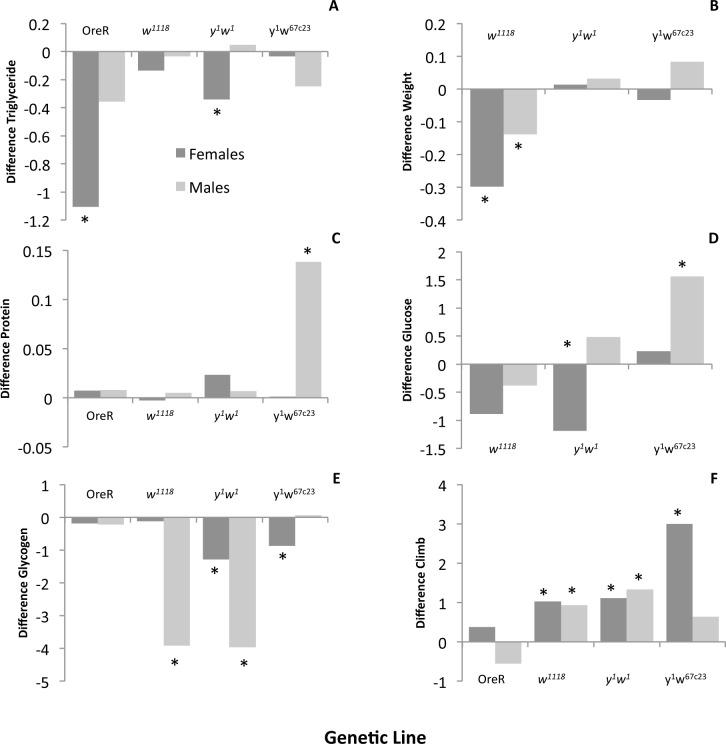
Sex and line specific effects of exercise on metabolic traits and climbing ability. Data from Study A. Y-axes indicate the difference in the mean phenotype between the exercise and control flies (positive value indicates increase with exercise, negative value indicates a decrease with exercise) per fly equivalent, units as in Fig 2. Females indicated in dark grey, males in light grey. * indicates a significant effect of exercise at p<0.05 as determined by a *post hoc* student's t-test. A. The exercised treatments showed significant sex-specific decreases in triglycerides in the females of two lines. B. Weight was significantly reduced by exercise in both sexes of the *w*^*1118*^ line. C. Protein significantly increased with exercise in males for line *y*^*1*^*w*^*67*^*c*^*23*^. D. Total glucose showed a significant decrease in female *y*^*1*^*w*^*1*^and increase in male *y*^*1*^*w*^*67*^*c*^*23*^ with exercise. E. Glycogen was significantly reduced in both males and females of *y*^*1*^*w*^*1*^, but only one sex of *w*^*1118*^ and *y*^*1*^*w*^*67*^*c*^*23*^ (males and females, respectively). F. Climbing ability showed a significant increase in both sexes of the *w*^*1118*^ and *y*^*1*^*w*^*1*^ lines, and females of *y*^*1*^*w*^*67*^*c*^*23*^ following exercise.

**Fig 4:**
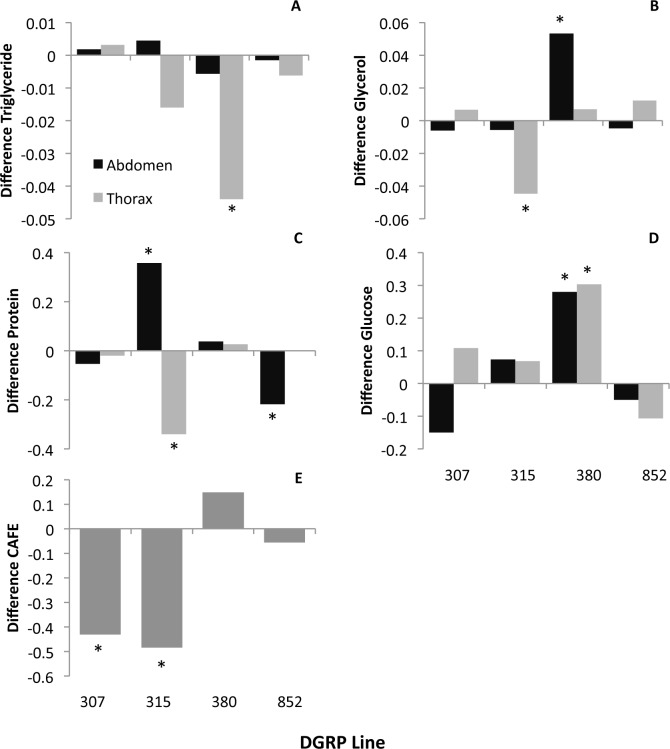
Exercise shows line- and tissue-specific effects on metabolic traits and line-specific effects on feeding behavior. Data from Study B. Y-axes indicate the relative difference in the mean phenotype between the exercise and control flies (positive value indicates increase with exercise, negative value indicates a decrease with exercise) per fly equivalent. Abdomen indicated in dark grey, thorax in light grey. * indicates a significant effect of exercise at p<0.05 as determined by a *post hoc* student's t-test. With exercise: A. Triglycerides exhibited a significant decrease in the thorax of a single line, 380; B. Glycerol was decreased in the thorax 315 and increased in the abdomen of 380; C. Protein showed a dramatic effect of line and tissue; D. Total glucose levels increased with exercise in line 380; and E. The amount of feeding as measured by the CAFE assay was decreased in line 307 and 315.

The second substantial finding of this study is that different genetic lines react to the exercise regime in distinct ways, indicating genetic variation for the effectiveness of exercise on various metabolic phenotypes. This finding is illustrated by the fact that Study A reveals significant line-by-exercise effects for all phenotypes except climbing performance (Table 1). It can also be seen in Fig 3, as for example only one of the four lines examined (*y*^*1*^*w*^*67c23*^) shows a strong increase in protein levels after exercise (Fig 3C). In addition, Study B demonstrates significant line-by-exercise effects for glucose and glycerol content (Table 2). This result is well illustrated in Fig 4D, where only line 380 shows a significant increase in glucose levels following exercise, while the other lines appear unchanged. Thus, our data demonstrate that genetic background is an important contributor to how individuals respond to exercise.

However, genetic background was not the only determinant to impact the outcomes of the exercise treatment. Sex also interacted with exercise treatment. In Study A, all exercise-induced phenotypes were impacted by sex, detected as a significant sex-by-treatment effect (Table 1). For example, only the females in lines OreR and *y*^*1*^*w*^*1*^ show a significant decrease in triglyceride levels after exercise, while the males of the same lines do not show this decrease (Fig 3A; see also S2 Fig). These results clearly demonstrate the importance of sex in the specific response to exercise observed.

While Study B was not specifically set up to test for the effect of age, for one of the physiological outcomes measured, data were collected at two different ages, before (3–5 days old) and after the exercise period (8–10 days old, Fig 4, Table 3). Adult flies between three and ten days old are generally considered to be fairly young, and over this young age range, the flies in Study B showed a significant increase in climbing ability (S3 Fig). However, these flies showed no overall effect of exercise (S3 Fig), meaning that there was no consistent improvement in climbing ability seen in flies under the moderate exercise regime, but that there was an increase in climbing ability from young (pre-exercise) to older (post-treatment). Work in other genotypes of flies have shown that flies older than those tested here (10–40 days) show an aging-related decline in climbing ability that can be partially rescued with exercise on the Power Tower in young flies [22]. Thus, these findings emphasize that future studies must consider ages of animals in their study design when assessing exercise effects.

**Table 3:** Effects of Motivation, Tissue, Exercise, and their interactions on metabolic traits and feeding behavior.

Phenotype	Motivation	Tissue	Treatment	Motivation * Tissue	Motivation * Treatment	Tissue * Treatment	Motivation * Tissue * Treatment
CAFÉ	*ns*	-	<0.05	-	*ns*	-	-
glucose	**<0.0001**	**<0.0001**	<0.05	*ns*	<0.05	*ns*	*ns*
glycerol	*ns*	**<0.01**	*ns*	**<0.01**	<0.05	*ns*	*ns*
protein	*ns*	<0.05	*ns*	**<0.0001**	*ns*	*ns*	*ns*
triglyceride	*ns*	<0.05	*ns*	*ns*	*ns*	*ns*	*ns*
weight	**<0.01**	-	*ns*	-	*ns*	-	-

ANOVA analysis, Bold indicates significance at a Bonferroni level, *ns*–not significant. Data from Study B.

### Motivation influences exercise effect on metabolic and fitness phenotypes

In preliminary studies, we found that some lines of flies would cease moving in response to the rotation of the TreadWheel after some period of time. In Study A, we dealt with this challenge by giving the flies a five-minute rest every 15–20 minutes which was adequate to allow them to resume exercise in the next bout (high intensity training). In Study B, rather than providing a rest period, the flies were stimulated to exercise for two hours continuously (moderate intensity training).

**Fig 5:**
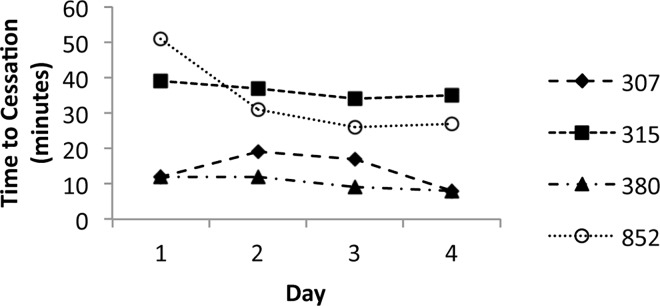
Time of activity during exercise is genotype dependent. Data from Study B. Duration of activity during exercise tended to decrease across the day of treatment with significant between the 852/315 lines and the 307/380 lines (p<0.0001), while no significant differences were found within the 852/315 and 307/380 line pairs. In subsequent analyses these lines were group as the low (307/380) and high (852/315) motivation.

Given the differences in exercise response across the four lines assayed in Study B (Table 2), we decided to investigate how long the flies of each genotype actually exercised. While the flies were continually stimulated to move for 2h/day during the exercise regime, the four different genotypes included in our study responded differently to this stimulation. Thus, we visually observed the duration of time during which the flies were actively moving up and down the vials (Fig 5). Using student t-tests we found that lines 852 and 315 exercised for significantly longer than 307 and 380 over the course of 4 days (p<0.0001). Our results indicate that these lines vary in the amount of time they exercise. Also of note is that motivation, as we defined it here, is not due primarily to fatigue. By two lines of evidence, we know the flies are not exhausted by the 10–50 minutes of exercise they exhibit under continuous gentle induction. First, the flies will resume exercise readily after receiving a short five-minute rest period as they did in Study A and, in studies using the Power Tower, using a more intense stimulus, the flies maintain high levels of continuous activity for two or more hours [22–24].

**Fig 6:**
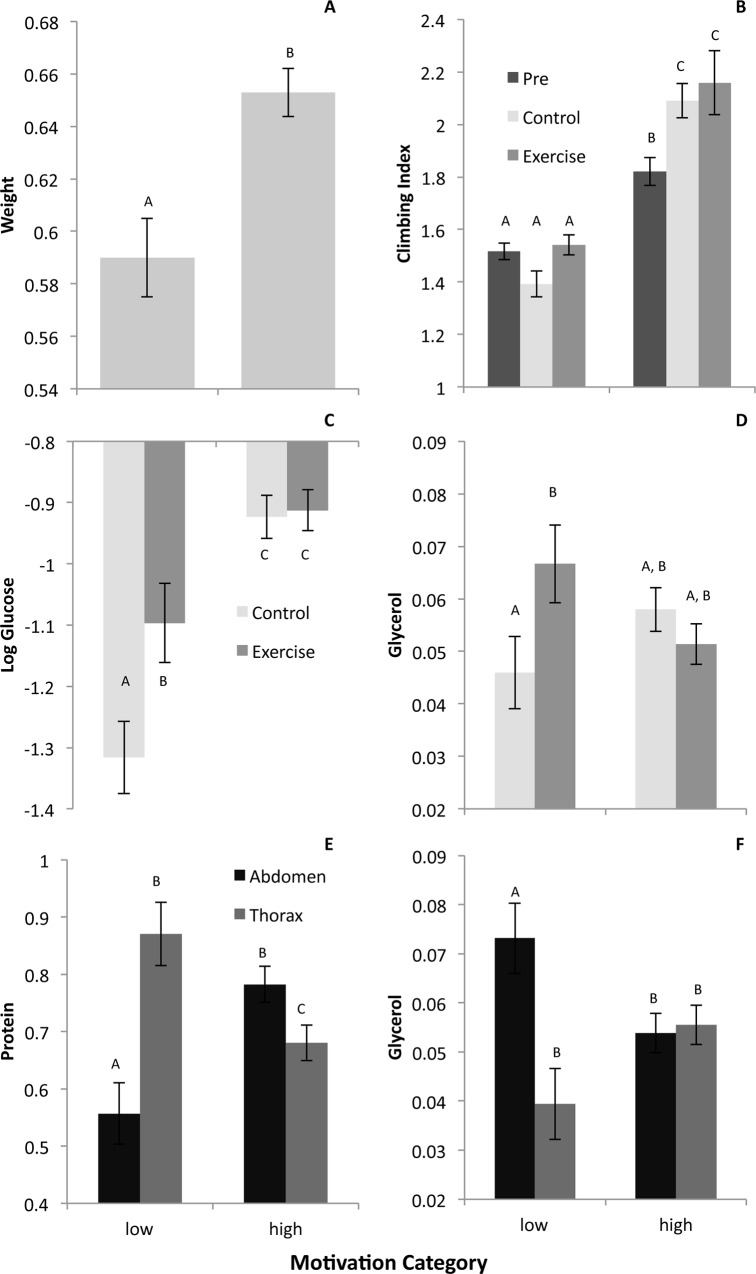
Motivation is correlated with baseline phenotypes and the effectiveness of exercise. Data from Study B. Units as in Fig 4. Error bars indicate one SEM. Within a graph, bars identified with different letters are statistically different from each other at p<0.05. A. Average weights were significantly higher in highly motivated flies. B. Climbing ability improved with age specifically in the high motivation flies. C. Total glucose levels were increased with exercise in the low motivation flies specifically, but high motivation lines maintained a high total sugar content independent of exercise. D. Exercised flies showed an increase in glycerol specifically in the low motivation flies. E. Tissue specific effects on glycerol were only observed in the low motivation flies. F. Baseline protein differed between tissues in the low and high motivation flies.

Next, we partitioned the DGRP lines into two groups based on motivation (low:307&380; high:315&852) to determine if motivation impacted their response to the exercise treatment. Partitioning the data this way, we discovered that several baseline measures differed between the high and low motivation DRGP lines (Fig 6, S5 Table). For example, average weights were significantly higher in highly motivated flies (Fig 6A), and climbing ability improved with age specifically in the high motivation flies (Fig 6B). Baseline protein differed between tissues in the low and high motivation flies, and tissue-specific effects on glycerol were only observed in the low motivation flies (Fig 6E & 6F). With respect to exercise, total glucose and glycerol levels were increased with exercise in the low motivation flies specifically, but high motivation lines maintained a higher total sugar content independent of exercise (Fig 6C & 6D). These findings suggest that motivation–or the inherent tendency of individuals to remain active–greatly influences a variety of physiological including responses to exercise.

### Feeding behavior interacts with exercise treatment

We used a modified version of the CAFE feeding assay [29] to measure food consumption in exercised and control flies. There was an overall effect of the exercise treatment on food consumption (Table 2 and 3) and an interaction between the exercise treatment and genotype (Table 2). However, perhaps counter intuitively, the largest variation in food consumption rates was observed in the control treatment, and two lines (307 and 315) significantly decreased their food consumption with the exercise treatment (Fig 4E, S6 Table). Food consumption also correlated with increased abdominal glucose and glycerol levels, as apparent from S4 Fig and the treatment interaction effects in S7 Table, a pattern driven by lines 307 and 315 primarily.

### Metabolic gene expression impacted by exercise

**Fig 7:**
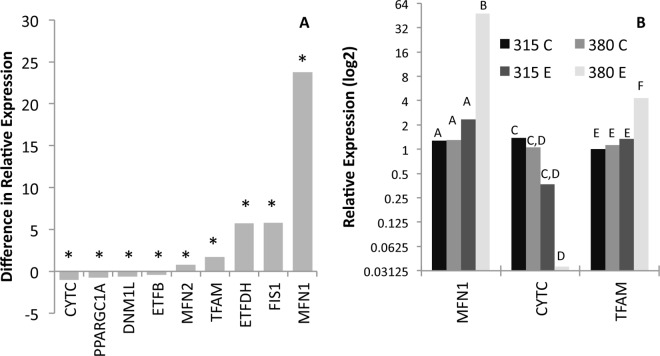
Significant impact of exercise and exercise-by-genotype differences in expression of mitochondrial genes. Data from Study B. Within a graph, bars identified with different letters are statistically different from each other at p<0.05. A. With exercise, four of the assayed genes showed a significant decrease in expression level, while five genes showed a significant increase. * indicates a significant effect of exercise at p<0.05 as determined by a *post hoc* student's t-test. B. The absolute amount of mRNA in three of the genes showed genotype-specific effects, with line 380 showing the most distinct pattern. For each gene, different letters indicate statistically significant differences by t-test at p<0.05, *i*.*e*. for MFN1, exercised flies from 380 differ from the other three groups. C-control; E-exercised.

Given the widespread impact of the TreadWheel-induced exercise on a range of metabolic and physiological phenotypes, we were interested in whether exercise influenced gene expression. Reports from several systems indicate that exercise can lead to significant changes to gene expression profiles[31]. Because genes associated with mitochondrial function and nutrient delivery are often detected as significantly changed in these studies [23,45–51], we focused on a panel of 13 genes involved in exercise-induced adaptions. With exercise, in lines DGRP 315 and 380, four of the assayed genes showed a significant decrease in expression level (*CYTC*, *PPARGC1A*, *DNM1L*, *ETFB*), while five genes showed a significant increase (*MFN2*, *TFAM*, *ETFDH*, *FIS1*, *MFN1*; Fig 7, Table 4 and S8 Table).

**Table 4:** Effects of Line, Exercise, and their interaction on expression of mitochondrial genes.

Human Gene (fly ortholog)	Gene fuction	Line	Treatment	Line * Treatment
MFN1 (fzo)	mitochondrial fusion	<0.01	**<0.001**	<0.01
MFN2 (Marf)	mitochondrial fusion	*ns*	<0.01	*ns*
FIS1 (Fis1)	mitochondrial fission	*ns*	**<0.01**	*ns*
OPA1 (Opa1)	mitochondrial fusion	*ns*	*ns*	*ns*
DNM1L(Drp1)	mitochondrial fission	*ns*	<0.01	*ns*
CYTC (Cyt-c-d)	electron transfer protein	<0.01	**<0.001**	<0.01
ETFDH (Etf-QO)	electron transfer protein	*ns*	**<0.001**	*ns*
ETFB (CG7834)	electron transfer protein	*ns*	<0.05	*ns*
TFAM (TFAM)	marker of mitochondrial biogenesis, associated with mitochondrial density	<0.05	**<0.01**	<0.05
PPARGC1A (srl)	transcriptional co-activator regulating genes involved in OXPHOS	*ns*	<0.05	*ns*
VEGFA / PDGFA (Pvf1)	angiogenesis and vasculature	*ns*	*ns*	*ns*
LDB3 (Zasp52)	sarcomere formation	*ns*	*ns*	*ns*
SDC (Sdc)	transmembrane receptor involved in energy homeostasis	*ns*	*ns*	*ns*

ANOVA analysis, Bold indicates significance at a Bonferroni level. *ns*–not significant Data from Study B.

The choreographic dynamics of mitochondrial fission and fusion play a critical role in maintaining cellular energy homeostasis, especially under stressful conditions such as exercise [52]. *TFAM* is an established marker of mitochondrial biogenesis and density[46] and showed a significant increase in expression in our exercised flies, implying that these flies have increased mitochondrial function. In addition, mitochondrial fusion is associated also with increased mitochondrial function, and the genes *MFN1* and *MFN2* (mitofusin proteins) [45] showed a corresponding increased expression with the exercise treatment. Also, *DNM1L*, which helps to mediate mitochondrial fission, showed an expected decrease in expression in the exercised flies; however, another mediator of mitochondrial fission, *FIS1*, showed a surprising increased in expression with exercise[53]. We also observed the puzzling pattern of decreased expression in three genes involved in electron transport and regulation of oxidative phosphorylation (*CYTC*, *ETFB*, and *PPARGC1A*), functions usually enhanced with improved mitochondrial performance [48].

The absolute amount of expression in three of the genes showed genotype-specific effects that interacted with exercise, with line 380 showing the most distinct pattern. Expression of *MFN1* and *TFAM* were significantly increased in exercised 380 flies, while *CYTC* was decreased relative to control flies. Line 380 was also the line that showed greater effect of exercise to decrease triglyceride storage, increase glycerol, and increase glucose levels. Conversely, line 315 showed no significant changes in expression of these three genes, and showed distinct metabolic responses to exercise from those observed in 380 flies with decreased glycerol, protein levels that differed between the thorax and abdomen, and reduced feeding. Two genotypes are not a sufficient sample size to make generalization about gene expression correlation with genotype-specific physiological response to exercise; however, the distinct differences between these two lines suggest that further exploration of this genotype-specific mito-metabolic phenotype correlation could be profitable.

The genotype-specific effects on mitochondrial gene function markers, as well as some of the counter-intuitive general findings of decreased expression of genes involved in electron transport and oxidative phosphorylation, suggests that the role of mitochondria in the exercise response in these flies is complex, and further study will be needed to understand the dynamics of mitochondrial response to exercise. However, the consistent increase in expression of genes associated with mitochondrial biogenesis and fusion shows that the TreadWheel, like other exercise systems, can induce gene expression changes in pathways that one would expect to be impacted. Taken together, these data suggest that TreadWheel-based exercise has an effect on mitochondrial function and metabolism, and moreover, these data reveal that some of the pathways involved in exercise-induced mitochondrial adaptions are conserved in flies. Future work to dissect how exercise interacts with genetic variation to influence mitochondrial dynamics will likely produce some new insights into the regulation of the mitochondrial network and its function.

## Conclusions

We have presented a novel method to exercise flies called the TreadWheel. Flies are intrinsically motived to move to the top of their enclosures, thus the TreadWheel induces low-impact exercise in adult *Drosophila* by slowly turning their enclosures. Our studies tested two five-day exercise regimes, one with short exercise bouts separated by short rest periods (Study A) in a cluster once a day and the other with a long continuous bout of moderate exercise once a day (Study B). We found that exercise through the short bout regime decreases body weight, total triglycerides, and glycogen and increases protein content and climbing performance. The short bout exercise regime had significantly varying impacts across genotype and sex for body weight, triglycerides, protein, glycogen, and glucose levels, indicating that studies of exercise effectiveness should consider these factors as well. We also found that the moderate exercise regime had variable effect across genetic lines for glycerol levels and feeding behavior. We suspect that the variation in line effects was in part due to the variation in motivation the distinct genotypes demonstrated. The moderate exercise study also demonstrated that a number of genes involved in mitochondrial and metabolic function change expression in response to exercise, showing that the molecular mechanisms involved in the exercise response in other systems also play a role in flies. Thus, the TreadWheel is a valuable tool for future high-throughput studies on how genotype interacts with exercise and other environmental factors like diet to influence the metabolic health and longevity of organisms. The ability to exercise Drosophila with a low stress regime will also lead to discoveries of which metabolic pathways are modified by exercise in this well characterized model organism.

## Supporting Information

S1 Fig Schematic for the negative geotaxis assay. A. Climbing assays were performed by placing a vial rack in front of a light box, with the camera placed 17cm in front of the rack. The rack was tapped down seven times at a rapid pace, and on the 7^th^ tap the camera, set on a 2 second timer, was activated. This process was repeated 4 times for each vial. B. Actual apparatus setup for climbing assays.
(TIFF)

S2 Fig Average Sex Specific Effect of Exercise on Metabolic Phenotypes. Data from Study A. Within a graph, bars identified with different letters are statistically different from each other at p<0.05. A. Both males and females showed reduced triglycerides with exercise. B. Females were the only sex with reduced weight with exercise. C. Only males gained a significant amount of protein with exercise. D. Only females showed reduced glucose levels with exercise. E. Both males and females showed reduced glycogen stores with exercise. F. Both males and females showed improved climbing performance with exercise.
(TIFF)

S3 Fig Line Specific Climbing Performance with Exercise in Study B. For two genetic lines (315 and 852) climbing performance improved with age (pre vs. control/exercise) indicated by *. However, there was not a significant improvement in climbing performance in control versus exercised flies.
(TIFF)

S4 Fig Positive correlation of glycerol and abdominal glucose levels with food consumption. Study B. A. Glycerol. B. Abdominal glucose. Color indicates treatment (red–control, blue- exercise), and data point shapes indicate tissue (circles abdomen, + thorax).
(TIFF)

S1 File TreadWheel design information. (DOCX)

S2 File Supplemental methods. (DOCX)

S1 Table Exercise regime used in Study A. (DOCX)

S2 Table Primers used in the Q-RT-PCR analyses. (DOCX)

S3 Table Summarizes all data for Study A. (DOCX)

S4 Table Data from Study B organized by line. (DOCX)

S5 Table Data from Study B organized by motivation category. (DOCX)

S6 Table Data from Study B organized by feeding behavior. (DOCX)

S7 Table ANOVA analysis of the feeding behavior data. (DOCX)

S8 Table Summarizes all gene expression data from the Q-PCR study. (DOCX)
