# Supplementary figures and images for: The TreadWheel: A Novel Apparatus to Measure Genetic Variation in Response to Gently Induced Exercise for Drosophila
Source: PLoS One. 2016 Oct 13;11(10):e0164706. doi: 10.1371/journal.pone.0164706 (PMC5063428; doi:10.1371/journal.pone.0164706)

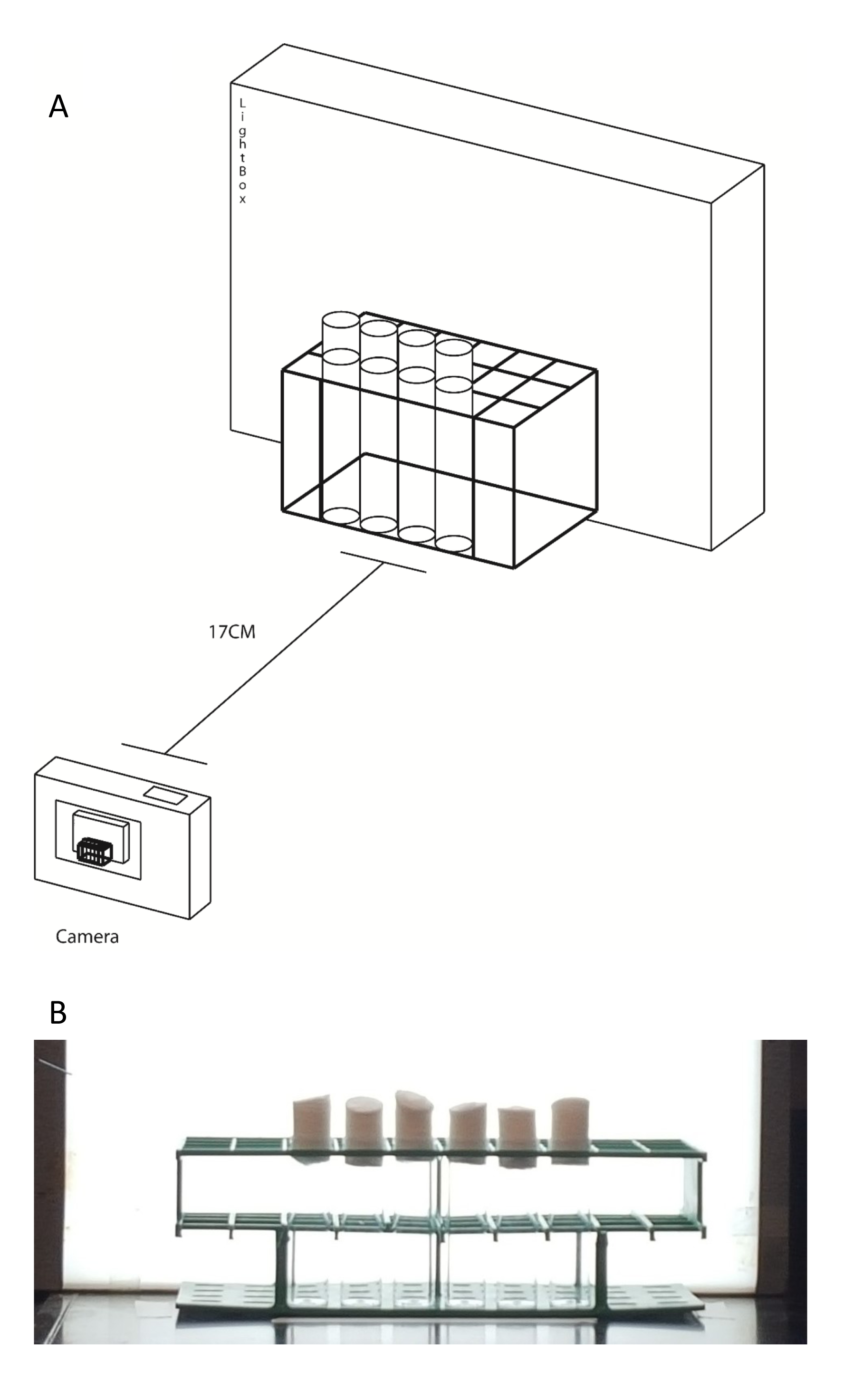

Supplement: S1 Fig — A. Climbing assays were performed by placing a vial rack in front of a light box, with the camera placed 17cm in front of the rack. The rack was tapped down seven times at a rapid pace, and on the 7th tap the camera, set on a 2 second timer, was activated. This process was repeated 4 times for each vial. B. Actual apparatus setup for climbing assays. (TIFF) [file pone.0164706.s001.tiff]

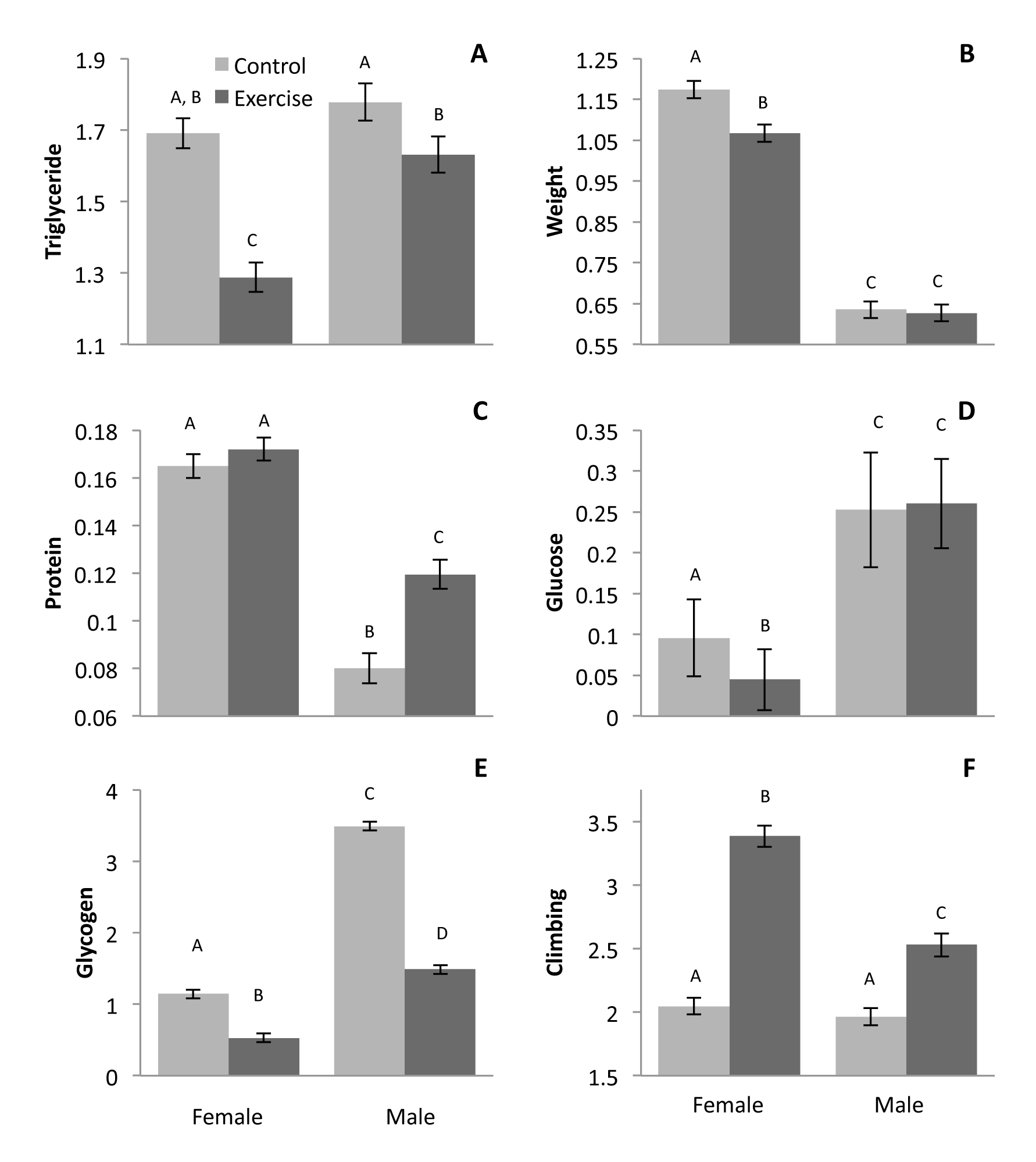

Supplement: S2 Fig — Data from Study A. Within a graph, bars identified with different letters are statistically different from each other at p<0.05. A. Both males and females showed reduced triglycerides with exercise. B. Females were the only sex with reduced weight with exercise. C. Only males gained a significant amount of protein with exercise. D. Only females showed reduced glucose levels with exercise. E. Both males and females showed reduced glycogen stores with exercise. F. Both males and females showed improved climbing performance with exercise. (TIFF) [file pone.0164706.s002.tiff]

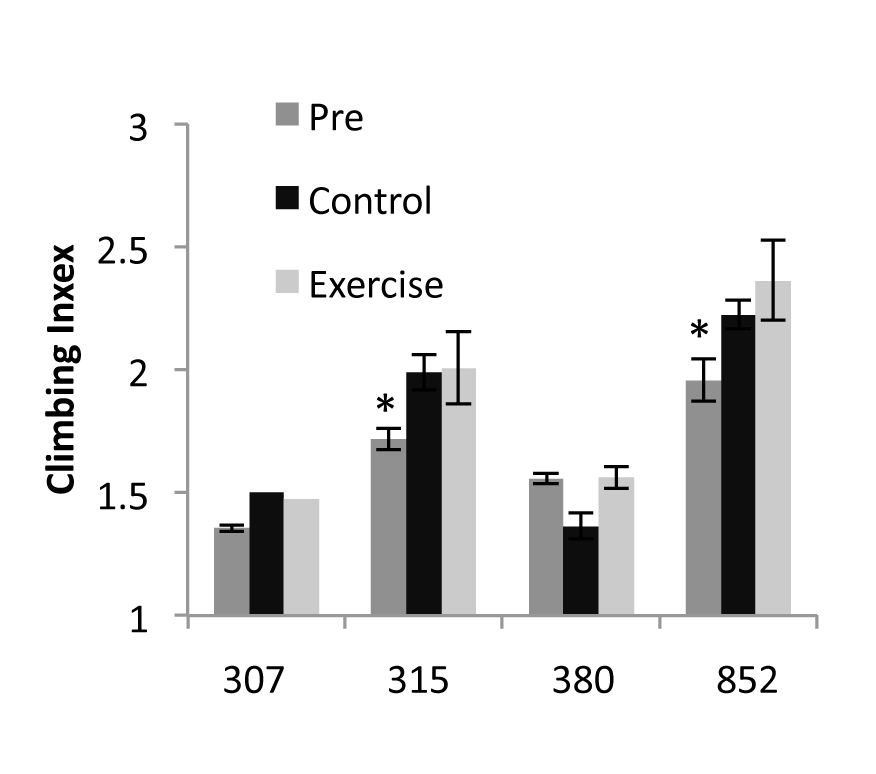

Supplement: S3 Fig — For two genetic lines (315 and 852) climbing performance improved with age (pre vs. control/exercise) indicated by *. However, there was not a significant improvement in climbing performance in control versus exercised flies. (TIFF) [file pone.0164706.s003.tiff]

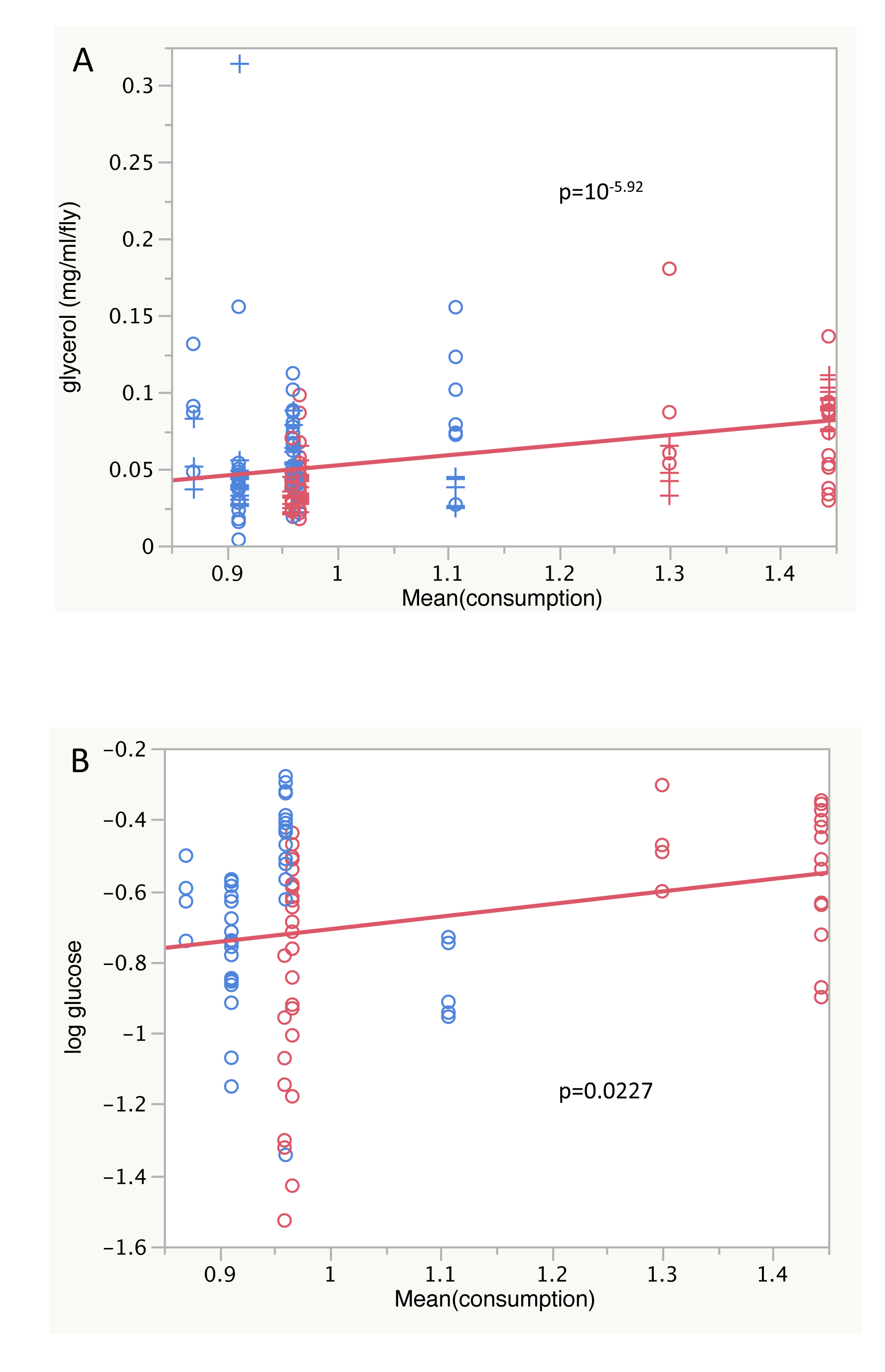

Supplement: S4 Fig — Study B. A. Glycerol. B. Abdominal glucose. Color indicates treatment (red–control, blue- exercise), and data point shapes indicate tissue (circles abdomen, + thorax). (TIFF) [file pone.0164706.s004.tiff]
