# Supplementary material for: The TreadWheel: A Novel Apparatus to Measure Genetic Variation in Response to Gently Induced Exercise for Drosophila
Source: PLoS One. 2016 Oct 13;11(10):e0164706. doi: 10.1371/journal.pone.0164706 (PMC5063428; doi:10.1371/journal.pone.0164706)
Supplement: S1 File — (DOCX) [file pone.0164706.s005.docx]

**TreadWheel Design and Construction Information**

TreadWheel non-custom parts list from McMaster-Carr

| **Part Name** | **Catalog Number** | **Quantity** |
| --- | --- | --- |
| Heavy Duty Vibration-Damping leveling Mount | 60855K71 | 4 |
| Stainless Steel Ball Bearing | 57155K306 | 8 |
| Plug-in Voltage Transformer (500MA, 120VAC input, 24 VAC output) | 70235K16 | 1 |
| Compact Square-Face DC Gear motor | 6409K23 | 1 |
| Tool Holder (clamps) | 1723A22 | 5 (10x) |
| 12L14 Carbon Steel Tight-Tolerance Rod | 5227T24 | 1 |
| Set Screw Shaft Collar | 6432K13 | 8 |
| Round-Belt Pulley | 6284K51 | 5 |
| Dart Controls – 25 Max RPM, Electric AC DC Motor | 13DV 1A | 1 |

**
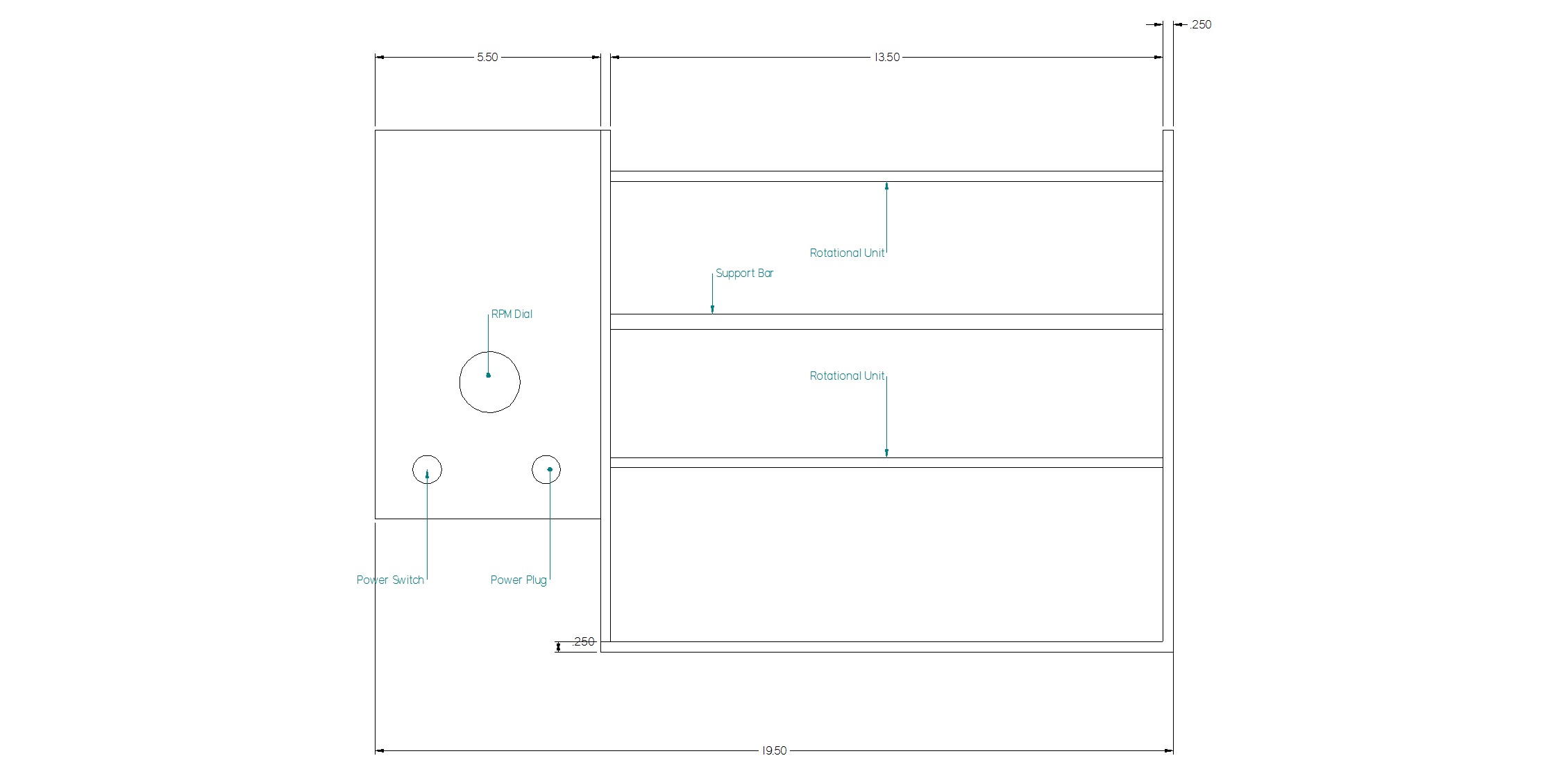
**

Front


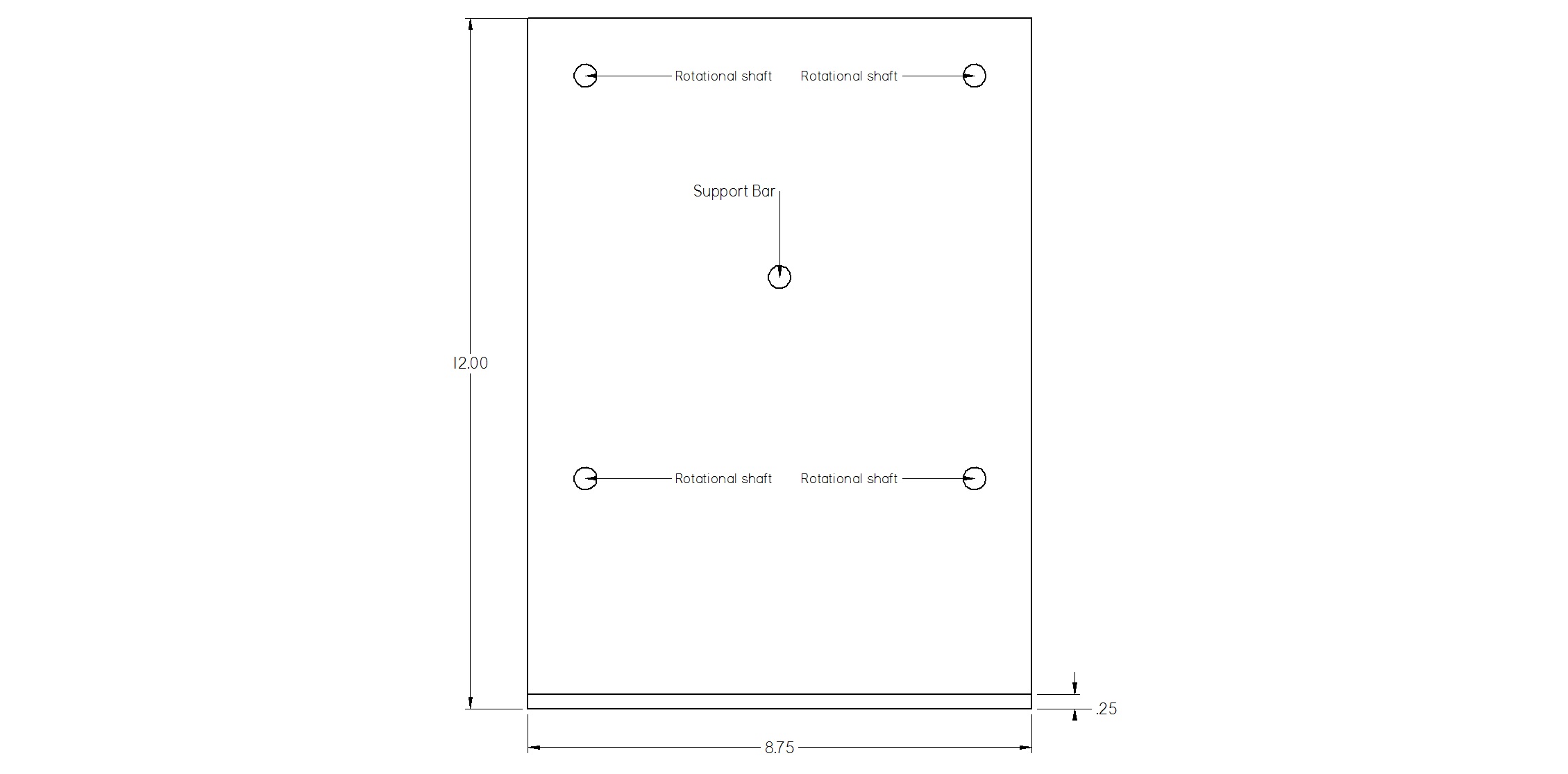


Side
