# Supplementary material for: The TreadWheel: A Novel Apparatus to Measure Genetic Variation in Response to Gently Induced Exercise for Drosophila
Source: PLoS One. 2016 Oct 13;11(10):e0164706. doi: 10.1371/journal.pone.0164706 (PMC5063428; doi:10.1371/journal.pone.0164706)
Supplement: S2 File — (DOCX) [file pone.0164706.s006.docx]

**Supplemental Methods**

**Glycogen and protein measurements (Study A)**

Glycogen contents were determined on pooled samples of 10 adult flies homogenized in 100μl H_2_O using the Sigma-Aldrich Glycogen Assay kit (MAK016) following manufacturer instructions. Protein content was determined using the Bradford Reagent from Amresco following manufacturer instructions on 5μl of adult fly homogenate for each metabolic assay (triglyceride, trehalose, and glycogen) diluted in 95μl of 0.15M NaCl. Triglyceride, trehalose, and glycogen measures were normalized to protein levels in the sample.

**Tissue Homogenization (Study B)**

To determine the impact of exercise on metabolites, flies were harvested 48h after cessation of the exercise protocol and stored at -20°C. For the metabolite analyses, abdomen and thorax tissues were analyzed separately. Dissections were performed on frozen flies; heads and legs were removed, and the fly bodies were separated into abdomens and thoraces. Tissue from five flies was pooled and homogenized in 75μl of iced lysis buffer (0.01M KH_2_PO_4_ and 1mM EDTA pH7.4). Samples were centrifuged at 0.4*g for 2 minutes at 4°C. Supernatants were stored at -20°C until further analysis. These supernatants are used for all the metabolite assays described below. To carry out the metabolite assays, all samples were thawed and then immediately placed on ice. All metabolite assays were performed in 96-well plates.

**Total Protein Content (Study B)**

To determine how protein content was affected by exercise, total protein content was estimated using the Lowry method [1]. 150μl of Lowry reagent (sequentially mix 0.5N NaOH, 2% Na_2_CO_3_, 2% Na_2_Tartrate, and 1% CuSO_4_-5H_2_O in a 10:1:1:100 ratio) and 10μl of sample was added into each well of a 96-well plate. The samples were mixed by incubation on an orbital shaker for 10 minutes at room temperature. 5μl of 1N folin was added to each well, and plates were incubated on a shaker for 20 minutes at room temperature. Absorbance at 630nm was recorded using a BioTek ELx8000 plate reader, and protein concentrations were calculated based on a standard curve of known protein concentrations.

**Glucose Content (Studies A and B)**

To determine the trehalose content of the fly extracts, the extracts were digested with trehalase overnight at 37c to gluclose. A master mix of 39.2ml glucose oxide peroxidase (Sigma, G3660-1CAP) and 0.8ml O-Dianisdine (Sigma, D2679) was made. In a 96-well plate, 250μl of reagent master mix and 1.67μl of sample were added to each well. Following a 3 minute incubation on an orbital shaker at room temperature, the plates were incubated at 37°C for 30 minutes. Plates were incubated for an additional 3 minutes on an orbital shaker at room temperature, and absorbance was measured at 540nm using a plate reader (BioTEK ELx800). Sample glucose concentrations were calculated based on a standard curve generated by measuring samples of known glucose concentration.

**Glycerol and triglycerides (Studies A and B)**

To determine the glycerol and triglyceride content of the fly extract, in each well of a 96-well plate, 200μl of free glycerol reagent (Sigma, F6428) and 2.5μl of fly extract were mixed. Plates were incubated on an orbital shaker at room temperature for 15 minutes. Then Initial Absorbance (IA) at 540nm was measured using a plate reader (BioTEK ELx800). 50μl of triglyceride reagent (Sigma, T2449) was added to each well, and plates were incubated on an orbital shaker at room temperature for 15 minutes. Following this second incubation, Final Absorbance (FA) at 540nm was recorded using a plate reader (BioTEK ELx800).

Computations: A = Absorbance, FA = Final Absorbance, IA = Initial Absorbance, Concentration of Standard = 2.5, F=0.80

Triglyceride concentrations (mg/ml) = (FA sample – (IA sample*F)/(FA standard – (IA blank*F)*Concentration of Standard

Glycerol concentrations (mg/ml) = (A Sample – A Blank)/(A Standard – A Blank)*Concentration of Standard

**Feeding behavior (Study B)**

This feeding assay is based on the CAFÉ (Capillary Feeder) assay developed by Ja and colleagues[2]. For this assay, pipette tips are inserted through foam vial tops, such that the glass capillaries can be stably held through the tops. A nutrient solution is provided through the capillaries to the flies. On the second day of exercise, five male virgin flies were loaded into each feeding vial. For lines 852 and 380, there were ten replicates each for treatment and control, for lines 315 and 380 there were eight replicates of each. 8μl of 10% sucrose, 5% yeast solution was loaded into each capillary. The height of liquid in each capillary was then individually measured with a ruler, and the capillary inserted into the vials through the pipette tips. Flies were acclimatized to the CAFÉ environment for one day. The capillaries were refilled and remeasured on the third day of exercise, and measurements were taken 8h later to determine the amount of food consumed.

**Climbing ability (Studies B)**

Climbing assays or negative geotaxis assays [3] were performed on the day before the exercise regime, and again following the 3^rd^ day of exercise in order to demonstrate the effect of regular exercise on climbing ability. 20 flies were loaded into each empty vial and placed in a rack placed in front of a light box (Figure 2; 852 n=3 for control and treatment, 315 n=4 for control and treatment, 307 n=1 for control and treatment, 380 n=7 for control and treatment). Flies were moved to the bottom of the vial by tapping the vial rack down seven times. On the 7^th^ tap, a camera with a 2 second delay timer was activated to record how high the flies could climb in 2 seconds. This process was repeated four times for each vial.

1. Lowry OH, Rosenbrough NJ, Farr AL, Randall RJ. Protein measurement with the Folin phenol reagent. Journal of Biological Chemistry. 1951;193: 265–275.

2. Ja WW, Carvalho GB, Mak EM, la Rosa de NN, Fang AY, Liong JC, et al. Prandiology of Drosophila and the CAFE assay. Proc Natl Acad Sci USA. 2007;104: 8253–8256. doi:10.1073/pnas.0702726104

3. Gargano JW, Martin I, Bhandari P, Grotewiel MS. Rapid iterative negative geotaxis (RING): a new method for assessing age-related locomotor decline in Drosophila. Experimental Gerontology. 2005;40: 386–395. doi:10.1016/j.exger.2005.02.005
