# Supplementary material for: The TreadWheel: A Novel Apparatus to Measure Genetic Variation in Response to Gently Induced Exercise for Drosophila
Source: PLoS One. 2016 Oct 13;11(10):e0164706. doi: 10.1371/journal.pone.0164706 (PMC5063428; doi:10.1371/journal.pone.0164706)
Supplement: S1 Table — (DOCX) [file pone.0164706.s007.docx]

**S1 Table. Short bout training exercise regime used in Study A.** Exercise and rest times in minutes used for each of the consecutive five days of exercise.

| Day | Bout 1 | Rest 1 | Bout 2 | Rest 2 | Bout 3 | Rest 3 | Bout 4 |
| --- | --- | --- | --- | --- | --- | --- | --- |
| 1 | 15 | 5 | 15 | 5 | 15 | 5 | 15 |
| 2 | 20 | 5 | 15 | 5 | 15 | 5 | 15 |
| 3 | 20 | 5 | 20 | 5 | 15 | 5 | 15 |
| 4 | 20 | 5 | 20 | 5 | 20 | 5 | 15 |
| 5 | 20 | 5 | 20 | 5 | 20 | 5 | 20 |
