# Supplementary material for: The TreadWheel: A Novel Apparatus to Measure Genetic Variation in Response to Gently Induced Exercise for Drosophila
Source: PLoS One. 2016 Oct 13;11(10):e0164706. doi: 10.1371/journal.pone.0164706 (PMC5063428; doi:10.1371/journal.pone.0164706)
Supplement: S2 Table — (DOCX) [file pone.0164706.s008.docx]

**S2 Table. Genes and Primers used in the Q-RT-PCR analysis.**

| Gene_PrimerName | Fly Gene Name | Human Gene Name | Primer |
| --- | --- | --- | --- |
| Dm CG12140 f | Electron transfer flavoprotein-ubiquinone oxidoreductase (Etf-QO) | electron transfer flavoprotein dehydrogenase (ETFDH) | TGAAAATGCACAGAGTTCGGAG |
| Dm CG12140 r | Electron transfer flavoprotein-ubiquinone oxidoreductase (Etf-QO) | electron transfer flavoprotein dehydrogenase (ETFDH) | ATAGTGGGTGGTTATCCTGGG |
| Dm CG12288 f | CG12288 | RNA binding motif protein 34 (RBM34) | GGACGAAGAAGGAGTTAAGCG |
| Dm CG12288 r | CG12288 | RNA binding motif protein 34 (RBM34) | TGATTGGCAAATTCCCCACGA |
| Dm rpl32 f | Ribosomal protein L32 (RpL32) | Ribosomal protein L32 (RPL32) | AGCATACAGGCCCAAGATCG |
| Dm rpl32 r | Ribosomal protein L32 (RpL32) | Ribosomal protein L32 (RPL32) | TGTTGTCGATACCCTTGGGC |
| Dm TBP f | TATA binding protein (Tbp) | TATA-box binding protein (TBP) | TAAGCCCCAACTTCTCGATTCC |
| Dm TBP r | TATA binding protein (Tbp) | TATA-box binding protein (TBP) | GCCAAAGAGACCTGATCCCC |
| Dm srl f | spargel (srl) | PPARG coactivator 1 alpha (PPARGC1A) | CTCTTGGAGTCCGAGATCCGCAA |
| Dm srl r | spargel (srl) | PPARG coactivator 1 alpha (PPARGC1A) | GGGACCGCGAGCTGATGGTT |
| Dm pvf1 f | PDGF- and VEGF-related factor 1 (Pvf1) | vascular endothelial growth factor A (VEGFA)/ platelet derived growth factor subunit A (PDGFA) | TGGAGCAGGCCGAGAACAAGTATT |
| Dm pvf1 r | PDGF- and VEGF-related factor 1 (Pvf1) | vascular endothelial growth factor A (VEGFA)/ platelet derived growth factor subunit A (PDGFA) | CCTGGACAATGAAGCGTTTGCGAT |
| Dm mfn2 f | Mitochondrial assembly regulatory factor (Marf) | mitofusion 2 (MFN2) | GAGACGACCACCTTTATCAACG |
| Dm mfn2 r | Mitochondrial assembly regulatory factor (Marf) | mitofusion 2 (MFN2) | GCCACCTTCATGTGATCCCG |
| Dm fzo f | fuzzy onions (fzo) | mitofusin 1 (MFN1) | TCAAATGGAAAAAGTGCCGTGA |
| Dm fzo r | fuzzy onions (fzo) | mitofusin 1 (MFN1) | TTACGTGCTCGGTTTCATTCG |
| Dm fis1 f | Fis1 | fission, mitochondrial 1 (FIS1) | GTCTGGCTTAAAATACTGCCGA |
| Dm fis 1 r | Fis1 | fission, mitochondrial 1 (FIS1) | CATACCCTTTGCCACTTCCTT |
| Dm opa1 f | Optic atrophy 1 (Opa1) | Optic atrophy 1 (OPA1) | TCAAGCTGCGATACATCGTCC |
| Dm opa1 r | Optic atrophy 1 (Opa1) | Optic atrophy 1 (OPA1) | GGCAGTCCATCCTTCCATTCC |
| Dm tfam f | mitochondrial transcription factor A (TFAM) | mitochondrial transcription factor A (TFAM) | CTTCGGCCTTCCTGCGATT |
| Dm tfam r | mitochondrial transcription factor A (TFAM) | mitochondrial transcription factor A (TFAM) | CTCCTTCTCGGAGTCGGAAAG |
| Dm drp1 f | Dynamin related protein 1 (Drp1) | dynamin 1-like (DNM1L) | ATCTACAGCCCACTCGATGAT |
| Dm drp1 r | Dynamin related protein 1 (Drp1) | dynamin 1-like (DNM1L) | GAAGCACTTCTTGGTGTGCAG |
| Dm CG7834 f | CG7834 | electron transfer flavoprotein beta subunit (ETFB) | AAA GCC GAC CTG GTC ATC CT |
| Dm CG7834 r | CG7834 | electron transfer flavoprotein beta subunit (ETFB) | CGG GGT CTT GGT CTT GAT TGT |
| Dm cytc-d f | Cytochrome c distal (Cyt-c-d) | cytochrome c, somatic (CYCS) | ATGGGTTCTGGTGATGCAGAG |
| Dm cytc-d r | Cytochrome c distal (Cyt-c-d) | cytochrome c, somatic (CYCS) | ACGACCCCGCCAAGATTTG |
| Dm zasp 52 f | Z band alternatively spliced PDZ-motif protein 52 (Zasp52) | LIM domain binding 3 (LDB3) | CCGAGCACACCGCCAGCCAA |
| Dm zasp 52 r | Z band alternatively spliced PDZ-motif protein 52 (Zasp52) | LIM domain binding 3 (LDB3) | CAACGCGGCCCGTCCCTTCTC |
| Dm Sdc f | Syndecan (Sdc) | syndecan 3 (SDC3) | CAG CAT CAT CGG CAA ACC AC |
| Dm Sdc r | Syndecan (Sdc) | syndecan 3 (SDC3) | CAC ACC CAC ATA CGC AGA GT |
|  |  |  |  |
