# Supplementary material for: The TreadWheel: A Novel Apparatus to Measure Genetic Variation in Response to Gently Induced Exercise for Drosophila
Source: PLoS One. 2016 Oct 13;11(10):e0164706. doi: 10.1371/journal.pone.0164706 (PMC5063428; doi:10.1371/journal.pone.0164706)
Supplement: S3 Table — (DOCX) [file pone.0164706.s009.docx]

**S3 Table. Summary of phenotypic data from Study A.** Ore R – Oregon R.

| Phenotype | Line | Sex | Treatment | Sample Size | Mean | Standard Error |
| --- | --- | --- | --- | --- | --- | --- |
| climbing | Ore R | female | control | 33 | 2.698 | 0.192 |
| climbing | Ore R | female | exercise | 27 | 3.072 | 0.327 |
| climbing | Ore R | male | control | 30 | 3.513 | 0.213 |
| climbing | Ore R | male | exercise | 30 | 2.960 | 0.430 |
| climbing | *w^1118^* | female | control | 33 | 2.480 | 0.219 |
| climbing | *w^1118^* | female | exercise | 33 | 3.509 | 0.264 |
| climbing | *w^1118^* | male | control | 39 | 1.866 | 0.181 |
| climbing | *w^1118^* | male | exercise | 30 | 2.798 | 0.328 |
| climbing | *y^1^w^1^* | female | control | 29 | 1.785 | 0.224 |
| climbing | *y^1^w^1^* | female | exercise | 30 | 2.895 | 0.180 |
| climbing | *y^1^w^1^* | male | control | 30 | 1.491 | 0.198 |
| climbing | *y^1^w^1^* | male | exercise | 30 | 2.831 | 0.384 |
| climbing | *y^1^w^67c23^* | female | control | 30 | 0.962 | 0.163 |
| climbing | *y^1^w^67c23^* | female | exercise | 30 | 3.954 | 0.296 |
| climbing | *y^1^w^67c23^* | male | control | 30 | 0.976 | 0.155 |
| climbing | *y^1^w^67c23^* | male | exercise | 33 | 1.618 | 0.170 |
| glucose | *w^1118^* | female | control | 9 | -1.774 | 0.366 |
| glucose | *w^1118^* | female | exercise | 10 | -2.662 | 0.386 |
| glucose | *w^1118^* | male | control | 9 | -0.537 | 0.245 |
| glucose | *w^1118^* | male | exercise | 9 | -0.921 | 0.203 |
| glucose | *y^1^w^1^* | female | control | 10 | -2.460 | 0.459 |
| glucose | *y^1^w^1^* | female | exercise | 10 | -3.649 | 0.214 |
| glucose | *y^1^w^1^* | male | control | 10 | -1.784 | 0.355 |
| glucose | *y^1^w^1^* | male | exercise | 10 | -1.302 | 0.357 |
| glucose | *y^1^w^67c23^* | female | control | 10 | -2.903 | 0.478 |
| glucose | *y^1^w^67c23^* | female | exercise | 10 | -2.669 | 0.487 |
| glucose | *y^1^w^67c23^* | male | control | 9 | -2.408 | 0.467 |
| glucose | *y^1^w^67c23^* | male | exercise | 10 | -0.843 | 0.220 |
| glycogen | Ore R | female | control | 10 | 0.918 | 0.069 |
| glycogen | Ore R | female | exercise | 10 | 0.734 | 0.063 |
| glycogen | Ore R | male | control | 10 | 3.185 | 0.168 |
| glycogen | Ore R | male | exercise | 10 | 2.965 | 0.096 |
| glycogen | *w^1118^* | female | control | 9 | 1.280 | 0.074 |
| glycogen | *w^1118^* | female | exercise | 10 | 1.155 | 0.044 |
| glycogen | *w^1118^* | male | control | 10 | 3.926 | 0.193 |
| glycogen | *w^1118^* | male | exercise | 10 | 0.019 | 0.003 |
| glycogen | *y^1^w^1^* | female | control | 11 | 1.293 | 0.073 |
| glycogen | *y^1^w^1^* | female | exercise | 10 | 0.010 | 0.002 |
| glycogen | *y^1^w^1^* | male | control | 10 | 3.981 | 0.173 |
| glycogen | *y^1^w^1^* | male | exercise | 10 | 0.018 | 0.003 |
| glycogen | *y^1^w^67c23^* | female | control | 10 | 1.084 | 0.056 |
| glycogen | *y^1^w^67c23^* | female | exercise | 12 | 0.214 | 0.136 |
| glycogen | *y^1^w^67c23^* | male | control | 10 | 2.872 | 0.222 |
| glycogen | *y^1^w^67c23^* | male | exercise | 10 | 2.935 | 0.187 |
| protein | Ore R | female | control | 6 | 0.155 | 0.009 |
| protein | Ore R | female | exercise | 8 | 0.162 | 0.004 |
| protein | Ore R | male | control | 4 | 0.060 | 0.002 |
| protein | Ore R | male | exercise | 4 | 0.068 | 0.009 |
| protein | *w^1118^* | female | control | 16 | 0.185 | 0.007 |
| protein | *w^1118^* | female | exercise | 14 | 0.182 | 0.006 |
| protein | *w^1118^* | male | control | 13 | 0.085 | 0.008 |
| protein | *w^1118^* | male | exercise | 9 | 0.090 | 0.004 |
| protein | *y^1^w^1^* | female | control | 10 | 0.158 | 0.011 |
| protein | *y^1^w^1^* | female | exercise | 8 | 0.181 | 0.011 |
| protein | *y^1^w^1^* | male | control | 8 | 0.113 | 0.004 |
| protein | *y^1^w^1^* | male | exercise | 9 | 0.119 | 0.008 |
| protein | *y^1^w^67c23^* | female | control | 11 | 0.162 | 0.011 |
| protein | *y^1^w^67c23^* | female | exercise | 11 | 0.163 | 0.019 |
| protein | *y^1^w^67c23^* | male | control | 5 | 0.062 | 0.003 |
| protein | *y^1^w^67c23^* | male | exercise | 7 | 0.201 | 0.019 |
| triglyceride | Ore R | female | control | 6 | 2.663 | 0.128 |
| triglyceride | Ore R | female | exercise | 8 | 1.557 | 0.095 |
| triglyceride | Ore R | male | control | 4 | 2.512 | 0.043 |
| triglyceride | Ore R | male | exercise | 4 | 2.156 | 0.318 |
| triglyceride | *w^1118^* | female | control | 16 | 1.403 | 0.070 |
| triglyceride | *w^1118^* | female | exercise | 14 | 1.267 | 0.049 |
| triglyceride | *w^1118^* | male | control | 13 | 1.718 | 0.071 |
| triglyceride | *w^1118^* | male | exercise | 9 | 1.685 | 0.055 |
| triglyceride | *y^1^w^1^* | female | control | 10 | 1.411 | 0.123 |
| triglyceride | *y^1^w^1^* | female | exercise | 8 | 1.071 | 0.051 |
| triglyceride | *y^1^w^1^* | male | control | 8 | 1.673 | 0.090 |
| triglyceride | *y^1^w^1^* | male | exercise | 9 | 1.721 | 0.085 |
| triglyceride | *y^1^w^67c23^* | female | control | 11 | 1.286 | 0.075 |
| triglyceride | *y^1^w^67c23^* | female | exercise | 11 | 1.254 | 0.048 |
| triglyceride | *y^1^w^67c23^* | male | control | 5 | 1.209 | 0.067 |
| triglyceride | *y^1^w^67c23^* | male | exercise | 7 | 0.963 | 0.055 |
| weight | *w^1118^* | female | control | 10 | 1.189 | 0.049 |
| weight | *w^1118^* | female | exercise | 9 | 0.891 | 0.020 |
| weight | *w^1118^* | male | control | 9 | 0.693 | 0.031 |
| weight | *w^1118^* | male | exercise | 9 | 0.554 | 0.015 |
| weight | *y^1^w^1^* | female | control | 10 | 1.094 | 0.027 |
| weight | *y^1^w^1^* | female | exercise | 10 | 1.106 | 0.033 |
| weight | *y^1^w^1^* | male | control | 11 | 0.609 | 0.018 |
| weight | *y^1^w^1^* | male | exercise | 11 | 0.640 | 0.039 |
| weight | *y^1^w^67c23^* | female | control | 10 | 1.239 | 0.060 |
| weight | *y^1^w^67c23^* | female | exercise | 10 | 1.206 | 0.039 |
| weight | *y^1^w^67c23^* | male | control | 10 | 0.603 | 0.019 |
| weight | *y^1^w^67c23^* | male | exercise | 11 | 0.686 | 0.047 |
