# Supplementary material for: The TreadWheel: A Novel Apparatus to Measure Genetic Variation in Response to Gently Induced Exercise for Drosophila
Source: PLoS One. 2016 Oct 13;11(10):e0164706. doi: 10.1371/journal.pone.0164706 (PMC5063428; doi:10.1371/journal.pone.0164706)
Supplement: S4 Table — (DOCX) [file pone.0164706.s010.docx]

**S4 Table. Summary of phenotypic data from Study B.**

| Phenotype | Line | Tissue | Treatment | Sample Size | Mean | Standard Error |
| --- | --- | --- | --- | --- | --- | --- |
| CAFÉ | 307 | - | control | 8 | 1.300 | 0.156 |
| CAFÉ | 307 | - | exercise | 8 | 0.870 | 0.100 |
| CAFÉ | 315 | - | control | 8 | 1.444 | 0.187 |
| CAFÉ | 315 | - | exercise | 8 | 0.960 | 0.142 |
| CAFÉ | 380 | - | control | 10 | 0.959 | 0.052 |
| CAFÉ | 380 | - | exercise | 10 | 1.107 | 0.068 |
| CAFÉ | 852 | - | control | 10 | 0.966 | 0.071 |
| CAFÉ | 852 | - | exercise | 10 | 0.911 | 0.055 |
| climbing | 307 | - | control | 1 | 1.500 | - |
| climbing | 307 | - | exercise | 2 | 1.471 | - |
| climbing | 307 | - | pre | 1 | 1.355 | 0.013 |
| climbing | 315 | - | control | 4 | 1.990 | 0.073 |
| climbing | 315 | - | exercise | 4 | 2.007 | 0.146 |
| climbing | 315 | - | pre | 8 | 1.718 | 0.043 |
| climbing | 380 | - | control | 4 | 1.364 | 0.053 |
| climbing | 380 | - | exercise | 4 | 1.560 | 0.044 |
| climbing | 380 | - | pre | 8 | 1.558 | 0.021 |
| climbing | 852 | - | control | 3 | 2.223 | 0.057 |
| climbing | 852 | - | exercise | 3 | 2.363 | 0.161 |
| climbing | 852 | - | pre | 6 | 1.957 | 0.085 |
| glucose | 307 | Abdomen | control | 4 | -0.467 | 0.062 |
| glucose | 307 | Abdomen | exercise | 4 | -0.617 | 0.050 |
| glucose | 307 | Thorax | control | 4 | -1.104 | 0.023 |
| glucose | 307 | Thorax | exercise | 4 | -0.996 | 0.067 |
| glucose | 315 | Abdomen | control | 13 | -0.552 | 0.053 |
| glucose | 315 | Abdomen | exercise | 18 | -0.478 | 0.056 |
| glucose | 315 | Thorax | control | 13 | -1.111 | 0.027 |
| glucose | 315 | Thorax | exercise | 19 | -1.042 | 0.012 |
| glucose | 380 | Abdomen | control | 8 | -1.147 | 0.082 |
| glucose | 380 | Abdomen | exercise | 7 | -0.867 | 0.041 |
| glucose | 380 | Thorax | control | 8 | -2.016 | 0.187 |
| glucose | 380 | Thorax | exercise | 6 | -1.713 | 0.132 |
| glucose | 852 | Abdomen | control | 20 | -0.730 | 0.057 |
| glucose | 852 | Abdomen | exercise | 19 | -0.779 | 0.037 |
| glucose | 852 | Thorax | control | 20 | -1.236 | 0.034 |
| glucose | 852 | Thorax | exercise | 19 | -1.342 | 0.055 |
| glycerol | 307 | Abdomen | control | 4 | 0.095 | 0.029 |
| glycerol | 307 | Abdomen | exercise | 4 | 0.089 | 0.017 |
| glycerol | 307 | Thorax | control | 4 | 0.047 | 0.007 |
| glycerol | 307 | Thorax | exercise | 4 | 0.053 | 0.010 |
| glycerol | 315 | Abdomen | control | 13 | 0.071 | 0.008 |
| glycerol | 315 | Abdomen | exercise | 18 | 0.065 | 0.006 |
| glycerol | 315 | Thorax | control | 13 | 0.094 | 0.003 |
| glycerol | 315 | Thorax | exercise | 19 | 0.049 | 0.004 |
| glycerol | 380 | Abdomen | control | 8 | 0.037 | 0.006 |
| glycerol | 380 | Abdomen | exercise | 7 | 0.090 | 0.016 |
| glycerol | 380 | Thorax | control | 8 | 0.030 | 0.003 |
| glycerol | 380 | Thorax | exercise | 6 | 0.037 | 0.004 |
| glycerol | 852 | Abdomen | control | 20 | 0.045 | 0.005 |
| glycerol | 852 | Abdomen | exercise | 19 | 0.040 | 0.007 |
| glycerol | 852 | Thorax | control | 20 | 0.039 | 0.003 |
| glycerol | 852 | Thorax | exercise | 19 | 0.051 | 0.015 |
| protein | 307 | Abdomen | control | 4 | 0.675 | 0.069 |
| protein | 307 | Abdomen | exercise | 4 | 0.620 | 0.073 |
| protein | 307 | Thorax | control | 4 | 0.830 | 0.057 |
| protein | 307 | Thorax | exercise | 4 | 0.811 | 0.079 |
| protein | 315 | Abdomen | control | 13 | 0.737 | 0.076 |
| protein | 315 | Abdomen | exercise | 18 | 1.094 | 0.041 |
| protein | 315 | Thorax | control | 13 | 0.935 | 0.072 |
| protein | 315 | Thorax | exercise | 19 | 0.596 | 0.051 |
| protein | 380 | Abdomen | control | 8 | 0.488 | 0.054 |
| protein | 380 | Abdomen | exercise | 7 | 0.527 | 0.038 |
| protein | 380 | Thorax | control | 8 | 0.893 | 0.137 |
| protein | 380 | Thorax | exercise | 6 | 0.919 | 0.068 |
| protein | 852 | Abdomen | control | 20 | 0.761 | 0.041 |
| protein | 852 | Abdomen | exercise | 19 | 0.544 | 0.048 |
| protein | 852 | Thorax | control | 20 | 0.628 | 0.043 |
| protein | 852 | Thorax | exercise | 19 | 0.630 | 0.063 |
| triglycerides | 307 | Abdomen | control | 4 | 0.096 | 0.012 |
| triglycerides | 307 | Abdomen | exercise | 4 | 0.098 | 0.016 |
| triglycerides | 307 | Thorax | control | 4 | 0.084 | 0.014 |
| triglycerides | 307 | Thorax | exercise | 4 | 0.087 | 0.005 |
| triglycerides | 315 | Abdomen | control | 13 | 0.080 | 0.003 |
| triglycerides | 315 | Abdomen | exercise | 18 | 0.084 | 0.005 |
| triglycerides | 315 | Thorax | control | 13 | 0.120 | 0.003 |
| triglycerides | 315 | Thorax | exercise | 19 | 0.104 | 0.007 |
| triglycerides | 380 | Abdomen | control | 8 | 0.073 | 0.007 |
| triglycerides | 380 | Abdomen | exercise | 7 | 0.067 | 0.004 |
| triglycerides | 380 | Thorax | control | 8 | 0.106 | 0.049 |
| triglycerides | 380 | Thorax | exercise | 6 | 0.062 | 0.006 |
| triglycerides | 852 | Abdomen | control | 20 | 0.063 | 0.004 |
| triglycerides | 852 | Abdomen | exercise | 19 | 0.061 | 0.006 |
| triglycerides | 852 | Thorax | control | 20 | 0.091 | 0.004 |
| triglycerides | 852 | Thorax | exercise | 19 | 0.085 | 0.008 |
| weight | 307 | - | control | 3 | 0.680 | 0.000 |
| weight | 307 | - | exercise | 3 | 0.640 | 0.020 |
| weight | 315 | - | control | 12 | 0.722 | 0.015 |
| weight | 315 | - | exercise | 18 | 0.704 | 0.013 |
| weight | 380 | - | control | 7 | 0.534 | 0.017 |
| weight | 380 | - | exercise | 5 | 0.584 | 0.004 |
| weight | 852 | - | control | 17 | 0.604 | 0.009 |
| weight | 852 | - | exercise | 18 | 0.602 | 0.013 |
