# Supplementary material for: The TreadWheel: A Novel Apparatus to Measure Genetic Variation in Response to Gently Induced Exercise for Drosophila
Source: PLoS One. 2016 Oct 13;11(10):e0164706. doi: 10.1371/journal.pone.0164706 (PMC5063428; doi:10.1371/journal.pone.0164706)
Supplement: S5 Table — (DOCX) [file pone.0164706.s011.docx]

**S5 Table. Summary of phenotypic data from Study B stratified by motivation.**

| Phenotype | Tissue | Treatment | Motivation | Sample Size | Mean | Standard Error |
| --- | --- | --- | --- | --- | --- | --- |
| café |  | control | low | 18 | 1.111 | 0.083 |
| café |  | control | high | 18 | 1.178 | 0.106 |
| café |  | exercise | low | 18 | 1.002 | 0.063 |
| café |  | exercise | high | 18 | 0.933 | 0.068 |
| climbing |  | control | low | 5 | 1.391 | 0.050 |
| climbing |  | control | high | 7 | 2.090 | 0.065 |
| climbing |  | exercise | low | 5 | 1.542 | 0.038 |
| climbing |  | exercise | high | 7 | 2.159 | 0.122 |
| climbing |  | Pre | low | 10 | 1.517 | 0.032 |
| climbing |  | Pre | high | 14 | 1.820 | 0.053 |
| glucose | Abdomen | control | low | 12 | -0.921 | 0.112 |
| glucose | Abdomen | control | high | 33 | -0.660 | 0.043 |
| glucose | Abdomen | exercise | low | 11 | -0.767 | 0.051 |
| glucose | Abdomen | exercise | high | 37 | -0.633 | 0.041 |
| glucose | Thorax | control | low | 12 | -1.712 | 0.178 |
| glucose | Thorax | control | high | 33 | -1.187 | 0.025 |
| glucose | Thorax | exercise | low | 10 | -1.426 | 0.142 |
| glucose | Thorax | exercise | high | 38 | -1.192 | 0.037 |
| glycerol | Abdomen | control | low | 12 | 0.056 | 0.013 |
| glycerol | Abdomen | control | high | 33 | 0.055 | 0.005 |
| glycerol | Abdomen | exercise | low | 11 | 0.090 | 0.011 |
| glycerol | Abdomen | exercise | high | 37 | 0.053 | 0.005 |
| glycerol | Thorax | control | low | 12 | 0.035 | 0.004 |
| glycerol | Thorax | control | high | 33 | 0.061 | 0.005 |
| glycerol | Thorax | exercise | low | 10 | 0.043 | 0.005 |
| glycerol | Thorax | exercise | high | 38 | 0.050 | 0.007 |
| protein | Abdomen | control | low | 12 | 0.550 | 0.049 |
| protein | Abdomen | control | high | 33 | 0.751 | 0.039 |
| protein | Abdomen | exercise | low | 11 | 0.561 | 0.036 |
| protein | Abdomen | exercise | high | 37 | 0.812 | 0.055 |
| protein | Thorax | control | low | 12 | 0.872 | 0.091 |
| protein | Thorax | control | high | 33 | 0.749 | 0.046 |
| protein | Thorax | exercise | low | 10 | 0.876 | 0.052 |
| protein | Thorax | exercise | high | 38 | 0.613 | 0.040 |
| triglyceride | Abdomen | control | low | 12 | 0.081 | 0.006 |
| triglyceride | Abdomen | control | high | 33 | 0.070 | 0.003 |
| triglyceride | Abdomen | exercise | low | 11 | 0.078 | 0.008 |
| triglyceride | Abdomen | exercise | high | 37 | 0.073 | 0.004 |
| triglyceride | Thorax | control | low | 12 | 0.099 | 0.033 |
| triglyceride | Thorax | control | high | 33 | 0.102 | 0.004 |
| triglyceride | Thorax | exercise | low | 10 | 0.072 | 0.006 |
| triglyceride | Thorax | exercise | high | 38 | 0.094 | 0.006 |
| weight |  | control | low | 10 | 0.578 | 0.025 |
| weight |  | control | high | 29 | 0.652 | 0.014 |
| weight |  | exercise | low | 8 | 0.605 | 0.012 |
| weight |  | exercise | high | 36 | 0.653 | 0.012 |
