# Supplementary material for: The TreadWheel: A Novel Apparatus to Measure Genetic Variation in Response to Gently Induced Exercise for Drosophila
Source: PLoS One. 2016 Oct 13;11(10):e0164706. doi: 10.1371/journal.pone.0164706 (PMC5063428; doi:10.1371/journal.pone.0164706)
Supplement: S6 Table — (DOCX) [file pone.0164706.s012.docx]

**S6 Table. Summary of phenotypic data from Study B stratified by feeding behavior.**

| Phenotype | Tissue | Treatment | CAFE cat | N Rows | Mean | Standard Error |
| --- | --- | --- | --- | --- | --- | --- |
| climb | - | Control | high | 10 | 1.753 | 0.081 |
| climb | - | Control | low | 14 | 1.712 | 0.091 |
| climb | - | Exercise | high | 10 | 1.788 | 0.092 |
| climb | - | Exercise | low | 14 | 1.835 | 0.103 |
| glycerol | Abdomen | Control | high | 17 | 0.077 | 0.009 |
| glycerol | Abdomen | Control | low | 28 | 0.043 | 0.004 |
| glycerol | Abdomen | Exercise | high | 22 | 0.070 | 0.006 |
| glycerol | Abdomen | Exercise | low | 26 | 0.054 | 0.008 |
| glycerol | Thorax | Control | high | 17 | 0.083 | 0.006 |
| glycerol | Thorax | Control | low | 28 | 0.037 | 0.002 |
| glycerol | Thorax | Exercise | high | 23 | 0.050 | 0.003 |
| glycerol | Thorax | Exercise | low | 25 | 0.048 | 0.011 |
| log glucose | Abdomen | Control | high | 17 | -0.532 | 0.043 |
| log glucose | Abdomen | Control | low | 28 | -0.849 | 0.059 |
| log glucose | Abdomen | Exercise | high | 22 | -0.503 | 0.048 |
| log glucose | Abdomen | Exercise | low | 26 | -0.800 | 0.030 |
| log glucose | Thorax | Control | high | 17 | -1.109 | 0.021 |
| log glucose | Thorax | Control | low | 28 | -1.459 | 0.088 |
| log glucose | Thorax | Exercise | high | 23 | -1.034 | 0.015 |
| log glucose | Thorax | Exercise | low | 25 | -1.431 | 0.060 |
| protein | Abdomen | Control | high | 17 | 0.722 | 0.060 |
| protein | Abdomen | Control | low | 28 | 0.677 | 0.041 |
| protein | Abdomen | Exercise | high | 22 | 1.008 | 0.053 |
| protein | Abdomen | Exercise | low | 26 | 0.539 | 0.036 |
| protein | Thorax | Control | high | 17 | 0.910 | 0.057 |
| protein | Thorax | Control | low | 28 | 0.704 | 0.053 |
| protein | Thorax | Exercise | high | 23 | 0.633 | 0.047 |
| protein | Thorax | Exercise | low | 25 | 0.699 | 0.056 |
| triglyceride | Abdomen | Control | high | 17 | 0.084 | 0.004 |
| triglyceride | Abdomen | Control | low | 28 | 0.066 | 0.003 |
| triglyceride | Abdomen | Exercise | high | 22 | 0.087 | 0.005 |
| triglyceride | Abdomen | Exercise | low | 26 | 0.063 | 0.004 |
| triglyceride | Thorax | Control | high | 17 | 0.111 | 0.005 |
| triglyceride | Thorax | Control | low | 28 | 0.095 | 0.014 |
| triglyceride | Thorax | Exercise | high | 23 | 0.101 | 0.006 |
| triglyceride | Thorax | Exercise | low | 25 | 0.079 | 0.007 |
| weight | - | Control | high | 15 | 0.713 | 0.013 |
| weight | - | Control | low | 24 | 0.583 | 0.010 |
| weight | - | Exercise | high | 21 | 0.695 | 0.012 |
| weight | - | Exercise | low | 23 | 0.598 | 0.010 |
