# Supplementary material for: The TreadWheel: A Novel Apparatus to Measure Genetic Variation in Response to Gently Induced Exercise for Drosophila
Source: PLoS One. 2016 Oct 13;11(10):e0164706. doi: 10.1371/journal.pone.0164706 (PMC5063428; doi:10.1371/journal.pone.0164706)
Supplement: S7 Table — (DOCX) [file pone.0164706.s013.docx]

**S7 Table.** **Effects of Feeding Behavior, Tissue, Exercise, and their interactions on metabolic traits.**

| Phenotype | CAFÉ | Tissue | Treatment | CAFE * Tissue | CAFE * Treatment | Tissue * Treatment | CAFE * Tissue * Treatment |
| --- | --- | --- | --- | --- | --- | --- | --- |
| glucose | *ns* | **<0.0001** | *ns* | *ns* | **<0.0001** | *ns* | *ns* |
| glycerol | **<0.01** | **<0.01** | <0.05 | <0.05 | *ns* | <0.05 | **<0.01** |
| protein | *ns* | ns | *ns* | *ns* | *ns* | *ns* | *ns* |
| triglyceride | *ns* | <0.05 | *ns* | *ns* | *ns* | *ns* | *ns* |
| weight | *ns* | - | **<0.0001** | - | *ns* | - | - |

Bold indicates significance at a Bonferroni level, *ns* – not significant. Data from Study B.
