# Supplementary material for: The TreadWheel: A Novel Apparatus to Measure Genetic Variation in Response to Gently Induced Exercise for Drosophila
Source: PLoS One. 2016 Oct 13;11(10):e0164706. doi: 10.1371/journal.pone.0164706 (PMC5063428; doi:10.1371/journal.pone.0164706)
Supplement: S8 Table — (DOCX) [file pone.0164706.s014.docx]

**S8 Table. Summary of Q-RT-PCR expression results by gene, genetic line, and treatment.** Gene name is given as human ortholog.

| Gene | Line | Treatment | Replicates | Mean | Standard Error |
| --- | --- | --- | --- | --- | --- |
| ETFDH | 315 | Control | 3 | 1.132 | 0.403 |
| ETFDH | 315 | Exercise | 3 | 3.904 | 0.715 |
| ETFDH | 380 | Control | 3 | 1.157 | 0.396 |
| ETFDH | 380 | Exercise | 3 | 9.850 | 2.456 |
| ETFB | 315 | Control | 3 | 1.090 | 0.328 |
| ETFB | 315 | Exercise | 3 | 0.776 | 0.055 |
| ETFB | 380 | Control | 3 | 1.041 | 0.213 |
| ETFB | 380 | Exercise | 3 | 0.532 | 0.074 |
| CYTC | 315 | Control | 3 | 1.383 | 0.769 |
| CYTC | 315 | Exercise | 3 | 0.366 | 0.071 |
| CYTC | 380 | Control | 3 | 1.051 | 0.226 |
| CYTC | 380 | Exercise | 3 | 0.035 | 0.003 |
| DNM1L | 315 | Control | 3 | 1.059 | 0.267 |
| DNM1L | 315 | Exercise | 3 | 0.570 | 0.033 |
| DNM1L | 380 | Control | 3 | 1.064 | 0.250 |
| DNM1L | 380 | Exercise | 3 | 0.357 | 0.082 |
| MFN1 | 315 | Control | 3 | 1.267 | 0.590 |
| MFN1 | 315 | Exercise | 3 | 2.353 | 0.132 |
| MFN1 | 380 | Control | 3 | 1.303 | 0.514 |
| MFN1 | 380 | Exercise | 3 | 47.786 | 6.923 |
| FIS1 | 315 | Control | 3 | 1.017 | 0.129 |
| FIS1 | 315 | Exercise | 3 | 3.224 | 0.245 |
| FIS1 | 380 | Control | 3 | 1.382 | 0.572 |
| FIS1 | 380 | Exercise | 3 | 10.785 | 1.922 |
| MFN2 | 315 | Control | 3 | 1.063 | 0.243 |
| MFN2 | 315 | Exercise | 3 | 1.422 | 0.273 |
| MFN2 | 380 | Control | 3 | 1.019 | 0.132 |
| MFN2 | 380 | Exercise | 3 | 2.171 | 0.350 |
| OPA1 | 315 | Control | 3 | 1.205 | 0.428 |
| OPA1 | 315 | Exercise | 3 | 1.307 | 0.071 |
| OPA1 | 380 | Control | 3 | 1.019 | 0.133 |
| OPA1 | 380 | Exercise | 3 | 1.396 | 0.239 |
| LDB3 | 315 | Control | 3 | 1.080 | 0.306 |
| LDB3 | 315 | Exercise | 3 | 1.052 | 0.177 |
| LDB3 | 380 | Control | 3 | 1.267 | 0.565 |
| LDB3 | 380 | Exercise | 3 | 0.594 | 0.101 |
| VEGFA / PDGFA | 315 | Control | 3 | 1.221 | 0.517 |
| VEGFA / PDGFA | 315 | Exercise | 3 | 1.089 | 0.178 |
| VEGFA / PDGFA | 380 | Control | 3 | 1.245 | 0.495 |
| VEGFA / PDGFA | 380 | Exercise | 3 | 1.046 | 0.241 |
| SDC | 315 | Control | 3 | 1.041 | 0.194 |
| SDC | 315 | Exercise | 3 | 1.692 | 0.338 |
| SDC | 380 | Control | 3 | 1.025 | 0.157 |
| SDC | 380 | Exercise | 3 | 0.997 | 0.131 |
| PPARGC1A | 315 | Control | 3 | 1.007 | 0.084 |
| PPARGC1A | 315 | Exercise | 3 | 0.671 | 0.032 |
| PPARGC1A | 380 | Control | 3 | 1.463 | 0.911 |
| PPARGC1A | 380 | Exercise | 3 | 0.320 | 0.015 |
| TFAM | 315 | Control | 3 | 1.009 | 0.100 |
| TFAM | 315 | Exercise | 3 | 1.328 | 0.089 |
| TFAM | 380 | Control | 3 | 1.123 | 0.355 |
| TFAM | 380 | Exercise | 3 | 4.286 | 0.618 |
